# Supplementary material for: Mechanism of kisspeptin neuron synchronization for pulsatile hormone secretion in male mice
Source: Cell Rep. Author manuscript; Available in PMC 2026 Mar 2. (PMC7618808; doi:10.1016/j.celrep.2022.111914)
Supplement: Supplemental information [file EMS212662-supplement-Supplemental_information.zip › 1-s2.0-S2211124722018137-mmc1.pdf]

Cell Reports, Volume *42*

Supplemental information

Mechanism of kisspeptin neuron synchronization for pulsatile hormone secretion in male mice

Su Young Han, Paul G. Morris, Jae-Chang Kim, Santosh Guru, Maria Pardo-Navarro, Shel-Hwa Yeo, H. James McQuillan, and Allan E. Herbison

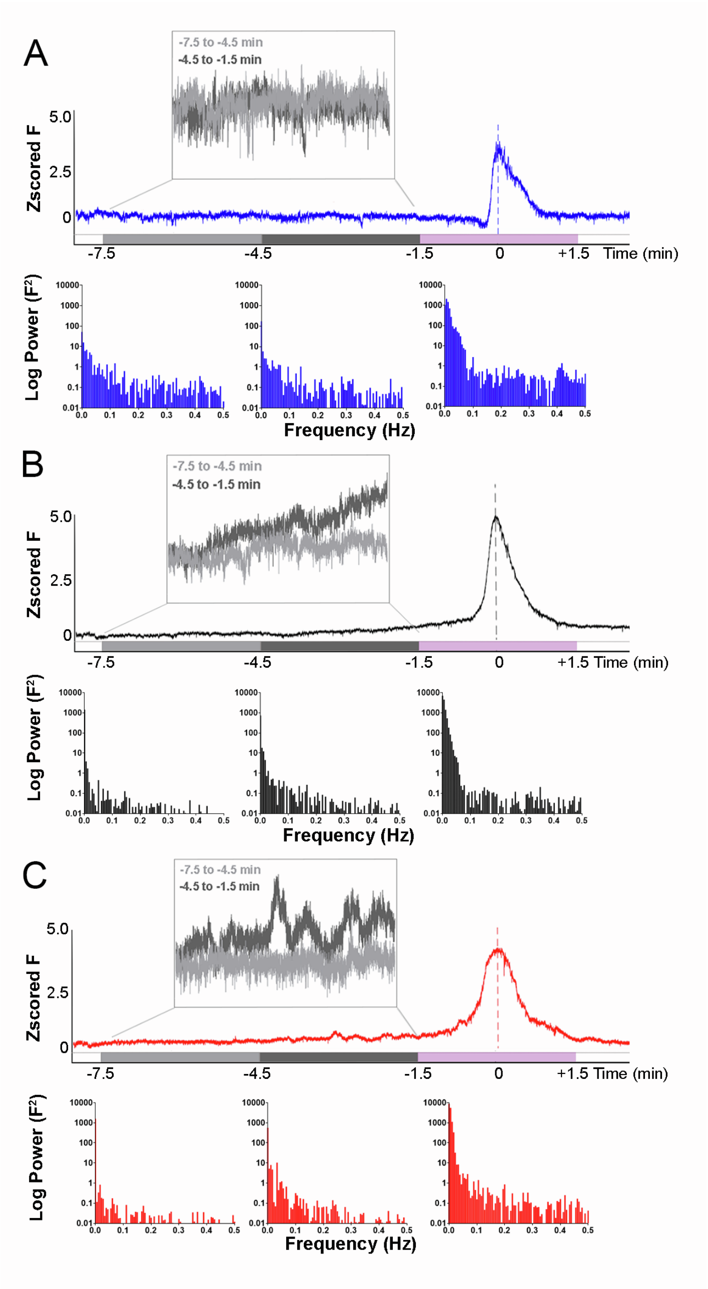

Suppl. Figure 1. *"Ramping" activity of individual ARN^{KISS} neurons in vivo*. **A-C,** GCaMP6 traces from three individual ARN^{KISS} neurons showing different patterns of activity leading up to a synchronization. Each section shows GCaMP6 levels (Z-scored F) in the 7.5 min period leading up to and including the abrupt increase in activity. The inset above shows overlapping fluorescence traces for the early 7.5-4.5 min (light grey) and close 4.5-1.5 (dark grey) min periods prior to a synchronization peak. Below, the power spectral density plot (log Power F^2) of slow frequency domain between 0-0.5 Hz for each 3 min bin showing the relative amounts of activity in the different frequency domains. In A, the activity spectra are very similar in the two blocks (7.5-4.5 and 4.5-1.5 min prior to synchronization) whereas for B and C there is an increase in low frequency activity during the -4.5 to -1.5 min blocks.

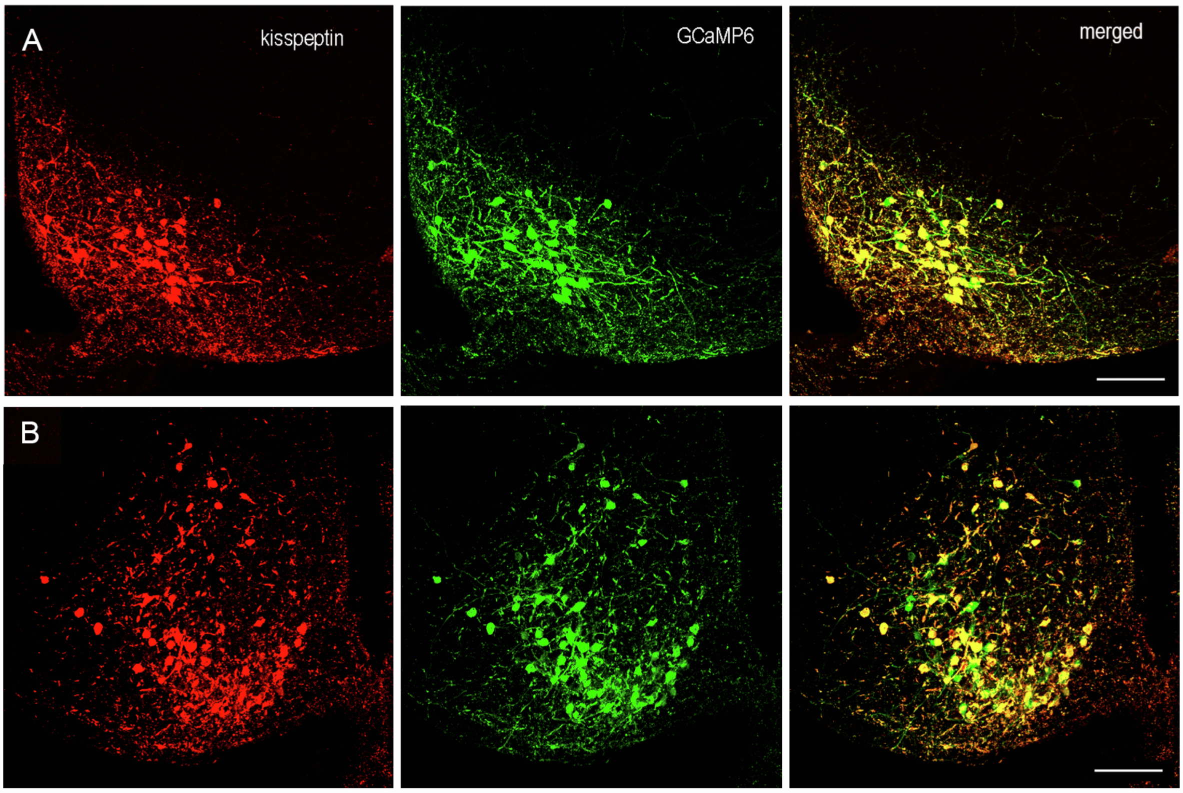

Suppl. Figure 2. *Characterization of Kiss1-Cre,Ai126D mice*. Dual label immunofluorescence at mid- (A) and caudal (B) coronal levels of the ARN showing the kisspeptin (red), GCaMP6 (green) and overlap of the two signals. Scale bars represent 100 μm.

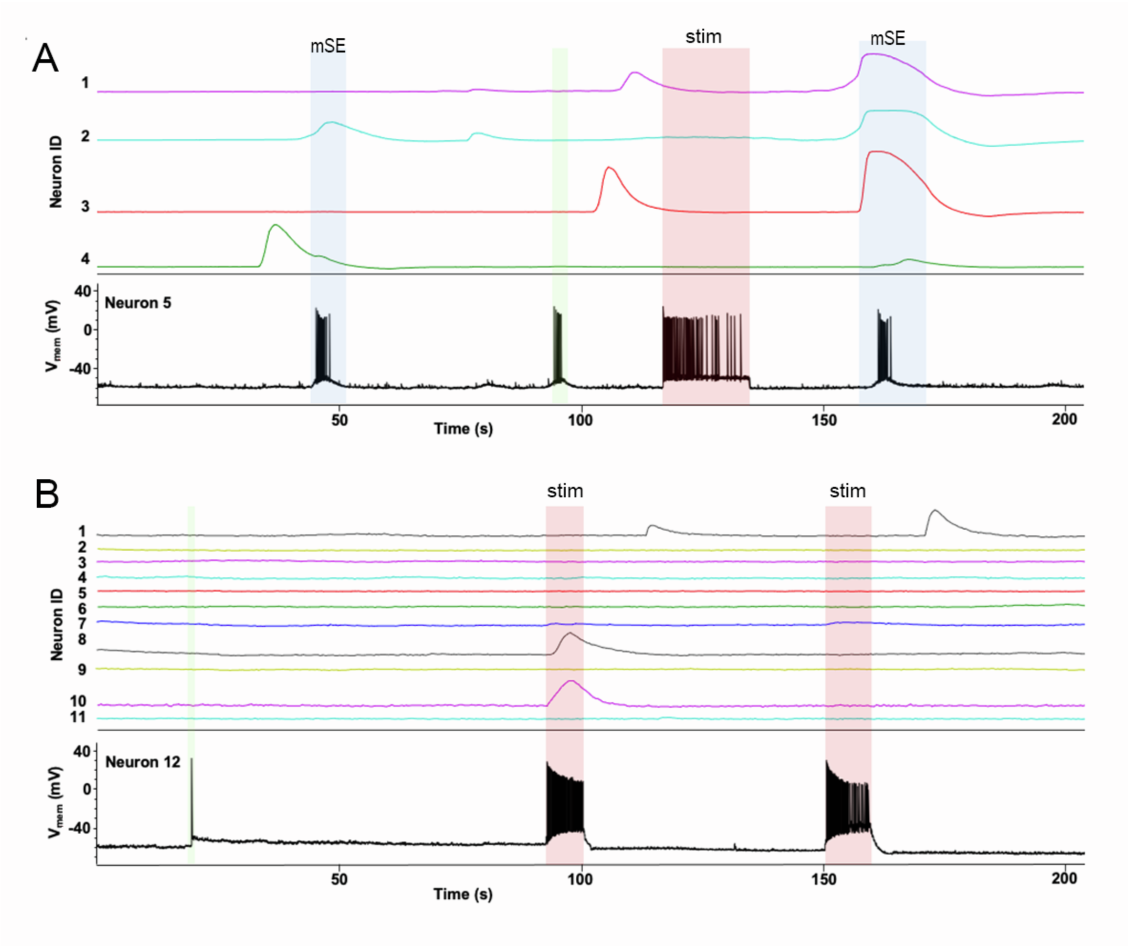

Suppl. Figure 3. *Effects of electrical activation of one ARN^{KISS} neuron on GCaMP activity of other ARN^{KISS} neurons in the brain slice.* **A,** GCaMP traces from 4 ARN^{KISS} neurons in the same slice as Neuron #5 (bottom trace) that was patched. Neuron #5 has three spontaneous episodes of burst firing with two associated with GCaMP increments other neurons (representing mSEs, blue) and one alone (green). However, artificial activation of Neuron #5 (pink) has no immediate effects on any of the other recorded cells. **B,** GCaMP traces from 11 neurons and a patched cell Neuron #12. Activation of firing in neuron #12 results in synchronized increases in GCaMP signal in neuron #8 and #10 on the first occasion but not subsequently.

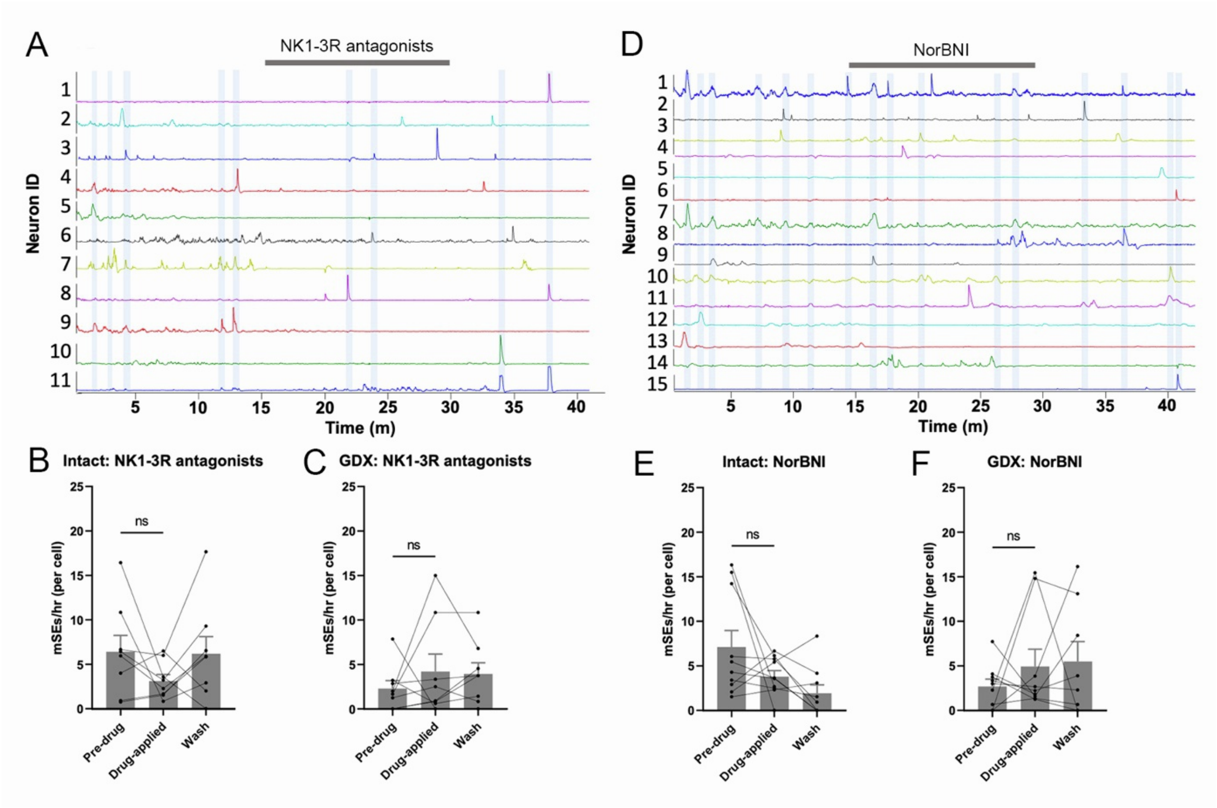

Suppl. Figure 4. *Tachykinin and dynorphin signaling are not essential for ARN^{KISS} neuron synchronization in vitro.* **A&D,** Recordings from brain slices showing the effect of 15 min exposure to the tachykinin receptor antagonist cocktail (1 μM SDZ-NKT 343, 1 μM GR94800, 3 μM SB 222200, intact male) or NorBNI (10 μM; GDX male) on GCaMP fluorescence recorded from 11 and 15 kisspeptin neurons. mSEs are indicated by blue shading. **B&C,** Histograms showing the individual data points and mean (+SEM) mSE rate for the pre-drug, drug-applied and wash periods in response to the tachykinin receptor antagonist cocktail in intact and GDX male mice brain slices. **E&F,** Histograms showing the individual data points and mean (+SEM) mSE rate for the pre-drug, drug-applied and wash periods in response to NorBNI in intact and GDX male mice brain slices.

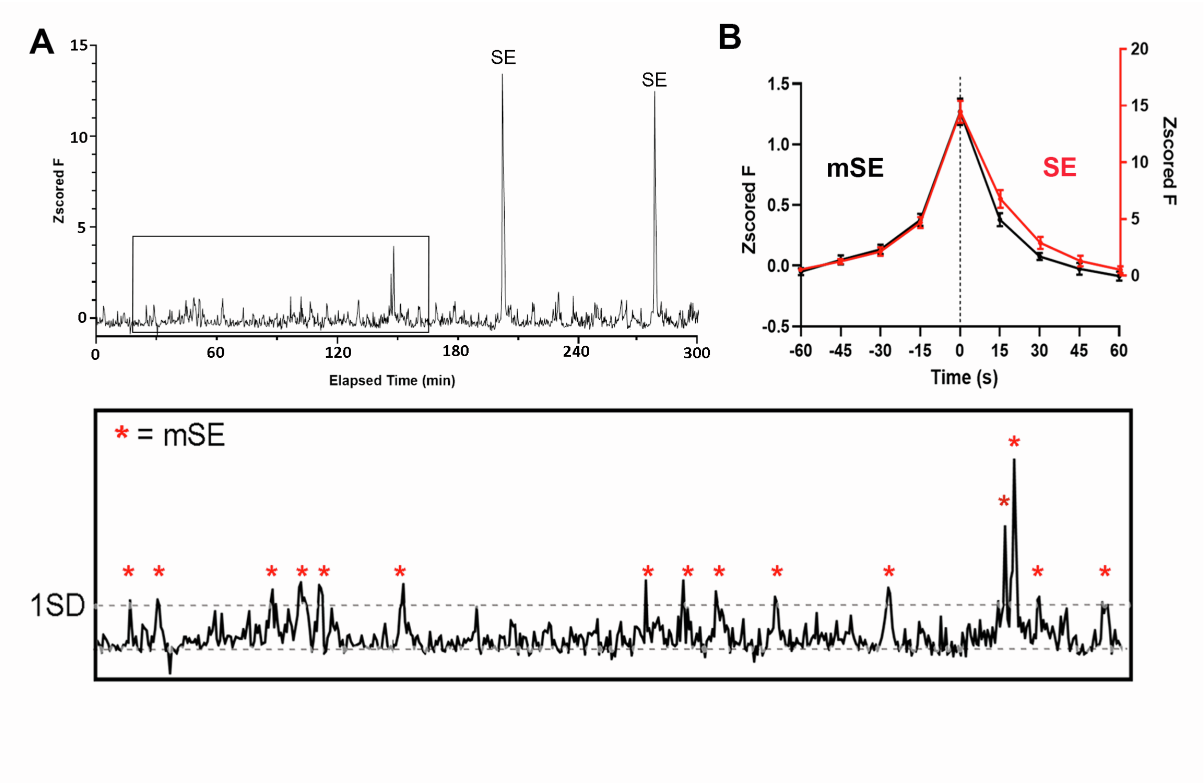

Suppl. Figure 5. *Low amplitude miniature SEs occur in vivo.* **A,** GCaMP fiber photometry recording from an intact male mouse showing the occurrence of two SEs. Baseline recording is expanded below to show frequent low-amplitude miniature synchronization events (mSEs, asterisks). Baseline and 1 standard deviation (SD) from mean is indicated by the dotted lines. **B,** Mean±SEM amplitude normalized profiles of SEs (red, 9 events) and mSEs (black, 269 events) recorded from intact male mice (N=5).

Suppl. Table 1. No changes occur in the dynamics of mSEs in the presence of antagonists for ionotropic glutamate receptors (CNQX + AP5), tachykinin receptors (TKR) or kappa opioid receptors (NorBNI).

	**Intact male**				**GDX male**			
	**Half width (s)**	**Rise time (s)**	**Decay time (s)**		**Half width (s)**	**Rise time (s)**	**Decay time (s)**	
**CNQX + AP5**				n=6				n=6
Pre-drug	13.8 ± 1.31	4.57 ± 0.436	6.52 ± 0.946		14.1 ± 1.11	4.51 ± 0.711	5.67 ± 0.523	
Drug	12.6 ± 1.61	3.65 ± 0.560	7.59 ± 0.704		20.4 ± 2.56	7.88 ± 2.28	7.44 ± 0.817	
**TKR antagonists**				n=7				n=5
Pre-drug	12.0 ± 1.84	4.00 ± 0.927	6.27 ± 0.420		13.6 ± 2.40	3.53 ± 0.730	6.73 ± 1.30	
Drug	10.1 ± 0.697	2.84 ± 0.584	6.65 ± 0.506		15.5 ± 2.64	5.23 ± 1.14	7.04 ± 0.347	
**NorBNI**				n=10				n=8
Pre-drug	12.9 ± 1.04	3.69 ± 0.288	6.43 ± 0.307		18.2 ± 3.23	5.51 ± 1.30	7.18 ± 0.795	
Drug	12.8 ± 1.15	4.18 ± 0.705	6.79 ± 0.624		13.8 ± 1.61	4.28 ± 0.842	6.12 ± 0.502	
**Cyclothiazide**				n=8				
Pre-drug	9.92 ± 0.89	3.56 ± 0.544	6.34 ± 0.423					
Drug	10.83 ± 0.75	3.01 ± 0.385	6.79 ± 0.624					
