# Supplementary material for: Mechanism of kisspeptin neuron synchronization for pulsatile hormone secretion in male mice
Source: Cell Rep. Author manuscript; Available in PMC 2026 Mar 2. (PMC7618808; doi:10.1016/j.celrep.2022.111914)
Supplement: Supplemental information [file EMS212662-supplement-Supplemental_information.zip › 1-s2.0-S2211124722018137-mmc3.pdf]

Cell Reports

Article

Mechanism of kisspeptin neuron synchronization for pulsatile hormone secretion in male mice

Graphical abstract

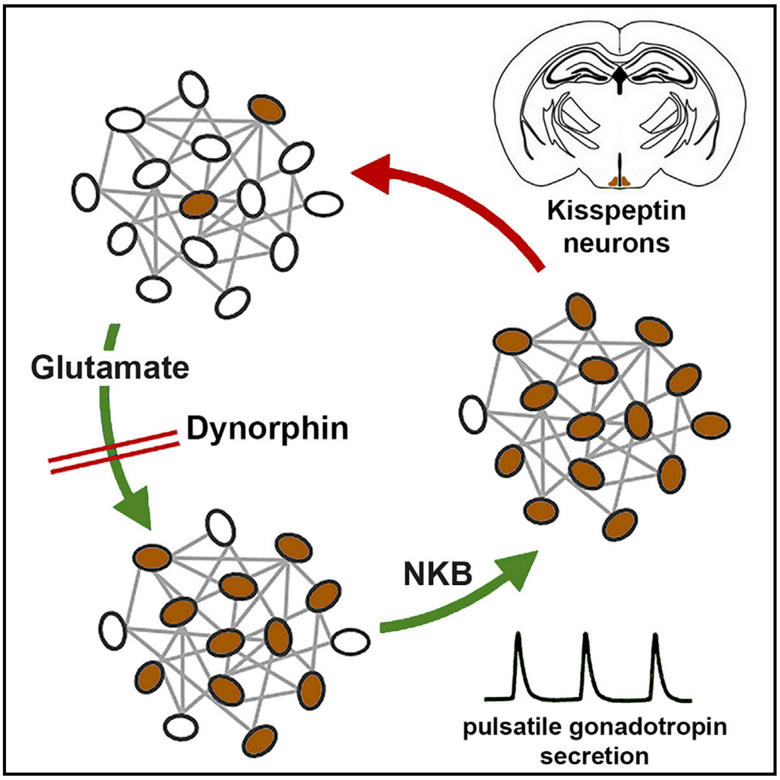

Highlights

- Arcuate kisspeptin neurons synchronize their activity in a non-linear manner
- Glutamate-AMPA signaling is critical for kisspeptin neuron synchronizations
- Neurokinin B transmission potentiates glutamate-driven synchronizations
- Dynorphin exerts state-dependent control of synchronization initiation

Authors

Su Young Han, Paul G. Morris, Jae-Chang Kim, ..., Shel-Hwa Yeo, H. James McQuillan, Allan E. Herbison

Correspondence

aeh36@cam.ac.uk

In brief

Hypothalamic kisspeptin neurons comprise the central pattern generator driving pulsatile reproductive hormone secretion in mammals. Han et al. show that the great majority of kisspeptin neurons exhibit brief periods of synchronized activity that are driven by glutamate transmission and modulated by the neuropeptides dynorphin and neurokinin B.

Han et al., 2023, Cell Reports *42*, 111914
January 31, 2023 © 2022 The Author(s).
https://doi.org/10.1016/j.celrep.2022.111914

CellPress

Cell Reports

CellPress OPEN ACCESS

Article

Mechanism of kisspeptin neuron synchronization for pulsatile hormone secretion in male mice

Su Young Han,[1,4] Paul G. Morris,[1,4] Jae-Chang Kim,[2] Santosh Guru,[1] Maria Pardo-Navarro,[1] Shel-Hwa Yeo,[1] H. James McQuillan,[3] and Allan E. Herbison[1,5,*]

[1]Department of Physiology, Development and Neuroscience, University of Cambridge, Cambridge CB2 3EG, UK

[2]Zurich Centre for Neuroeconomics, Department of Economics, University of Zurich, 8006 Zurich, Switzerland

[3]Centre for Neuroendocrinology and Department of Physiology, University of Otago School of Biomedical Sciences, Dunedin 9054, New Zealand

[4]These authors contributed equally

[5]Lead contact

*Correspondence: aeh36@cam.ac.uk

https://doi.org/10.1016/j.celrep.2022.111914

SUMMARY

The mechanism by which arcuate nucleus kisspeptin (ARN^{KISS}) neurons co-expressing glutamate, neurokinin B, and dynorphin intermittently synchronize their activity to generate pulsatile hormone secretion remains unknown. An acute brain slice preparation maintaining synchronized ARN^{KISS} neuron burst firing was used alongside *in vivo* GCaMP GRIN lens microendoscope and fiber photometry imaging coupled with intra-ARN microinfusion. Studies in intact and gonadectomized male mice revealed that ARN^{KISS} neuron synchronizations result from near-random emergent network activity within the population and that this was critically dependent on local glutamate-AMPA signaling. Whereas neurokinin B operated to potentiate glutamate-generated synchronizations, dynorphin-kappa opioid tone within the network served as a gate for synchronization initiation. These observations force a departure from the existing "KNDy hypothesis" for ARN^{KISS} neuron synchronization. A "glutamate two-transition" mechanism is proposed to underlie synchronizations in this key hypothalamic central pattern generator driving mammalian fertility.

INTRODUCTION

Understanding the mechanisms through which neural networks generate synchronized patterns of activity remains a key issue in neurobiology.[1] While oscillatory behavior is common, it is exemplified by brainstem central pattern generators controlling fundamental processes such as breathing and locomotion. Despite the well-established presence of pacemaker neurons and recurrent inhibition within such networks, the precise operation of central pattern generators is only beginning to be deciphered.[2]

Fertility in mammals is critically dependent upon a hypothalamic central pattern generator that drives the episodic release of gonadotropin-releasing hormone (GnRH) responsible for pulsatile reproductive hormone secretion.[3,4] Recent studies in mice have identified that a small population of ∼2,000 kisspeptin neurons located in the arcuate nucleus (ARN) is the central pattern generator driving this ultradian rhythm. The selective optogenetic activation of ARN kisspeptin (ARN^{KISS}) neurons generates increments in luteinizing hormone (LH) almost indistinguishable from endogenous LH pulses,[5] while optogenetic inhibition of these cells suppresses, and then re-sets, pulsatile LH secretion.[6] Furthermore, GCaMP photometry recordings in freely behaving male and female mice demonstrate an abrupt and robust increase in ARN^{KISS} neuron population activity immediately prior to each LH pulse.[6–8] These synchronization events are reminiscent of multi-unit activity recordings made from cells in the ARN of the monkey[9] and goat[10] and suggest that the ARN^{KISS} neurons represent a highly conserved mammalian GnRH pulse generator.[4,11]

The key unresolved question in GnRH pulse generation is how the ARN^{KISS} neurons synchronize their activity to generate repetitive bursts of activity that drive pulsatile hormone release. At present, the Kisspeptin-Neurokinin B-Dynorphin (KNDy) hypothesis prevails. This arose from the observation that ARN^{KISS} neurons co-express neurokinin B (NKB) and dynorphin in almost all mammals[12,13] and experiments in mice showing that essentially all ARN^{KISS} neurons synthesize tachykinin and kappa opioid receptors that activate and suppress their firing, respectively.[14,15] Thus, the "KNDy hypothesis" posits that reciprocal interconnections between ARN^{KISS} neurons using tachykinin and dynorphin signaling operate sequentially to activate and then terminate synchronized network activity.[16–18] While an attractive hypothesis, technical limitations have precluded direct confirmation of the KNDy hypothesis being responsible for spontaneous pulse generation *in vitro* or *in vivo*.

We have used here GCaMP miniscopes to determine the detailed activity patterns of individual ARN^{KISS} neurons across

© 2022 The Author(s).
This is an open access article under the CC BY license (http://creativecommons.org/licenses/by/4.0/).

synchronization episodes in freely behaving mice. This revealed a remarkable degree of functional homogeneity within the ARN^{KISS} neuron population despite synchronizations involving the non-linear recruitment of individual cells. To explore this further, we developed a novel *in vitro* brain slice preparation that maintained spontaneous synchronized activity among the ARN^{KISS} neurons. These synchronizations exhibited semi-random emergent network behavior dependent upon glutamate signaling through AMPA receptors. Returning to freely behaving mice, microinfusions of antagonists into the ARN while simultaneously recording ARN^{KISS} neuron activity with GCaMP photometry demonstrated the critical role of glutamate in synchronization initiation *in vivo* with tachykinins only modulating the amplitude of these events. Kappa opioids were not found to control the termination of ARN^{KISS} neuron synchronizations but, rather, operated as a state-dependent gate for their initiation. These observations demonstrate that contrary to the KNDy hypothesis, the ARN^{KISS} neuron synchronization events underpinning mammalian fertility arise from emergent glutamate-based network activity that is facilitated or gated by neuropeptide transmission.

RESULTS

Heterogeneous recruitment of ARN^{KISS} neurons for synchronization events *in vivo*

Gradient-index (GRIN) lens recordings (UCLA Miniscope) were made from AAV9-FLEX-GCaMP6s-injected *Kiss1*-Cre male mice. Prior studies have demonstrated that ~60% of ARN^{KISS} neurons express GCaMP6 and that ~90% of GCaMP-expressing cells are immunoreactive for kisspeptin in this model.[6] Animals were gonadectomized (GDX) so as to generate a state in which ARN^{KISS} neuron synchronizations driving pulsatile LH secretion occur approximately every 10 min.[8] Fluorescent signals were obtained from 13 to 50 neurons in individual mice (n = 5). Abrupt, synchronized increases in GCaMP fluorescence were recorded from almost all ARN^{KISS} neurons, although each cell did not always participate in every synchronization (114 cells, 23 synchronizations) (Figures 1A and 1B) (Video S1). On average, 81% of ARN^{KISS} neurons contributed to each synchronization event (SE) but did so in an unpredictable manner with on-going SEs (Figure 1A). The inter-SE interval was 9.2 ± 3.3 min, which is identical to the interval observed between population SEs measured using GCaMP fiber photometry under the same conditions (8.9 ± 0.5 min).[6] The duration of each individual activation ranged from 30 to 130 s with the mean half maximum of the rising and falling phase being 9 ± 3 s and 15 ± 5 s, respectively (Figure 1C).

Detailed assessments of shifts in GCaMP fluorescence using Fast Fourier Transformation demonstrated that 33% ± 18% of neurons exhibited a gradual rise in baseline activity up to 3 min prior to the abrupt increase in signal associated with the population SE (Figure S1A). Whereas most neurons have a steady baseline rate of activity (Figure S1A), others exhibit a gradual rise in activity (Figure S1B) or oscillatory activity (Figure S1C) in the 3-min period before SE onset. Cells exhibiting these "ramping" patterns of activity did not necessarily show the same pattern of activity for each SE they were involved in.

The time of the peak fluorescence signal was found to vary by up to 20 s among neurons contributing to an SE (Figure 1D). Although the order of neurons contributing to repeated SEs was not predictable, a moderate correlation (Spearman coefficient 0.52 ± 0.06) existed for those neurons activated first during one SE to be among those cells that were activated first in a subsequent SE (Figures 1E and 1F). For example, a cell activated first during one SE could sometimes be among the first three cells activated on a subsequent SE (Figures 1E and 1F). Equally, however, a cell activated first during one SE could be among the last cells activated in subsequent SEs (Figures 1E and 1F). Cells activated first during an SE did not necessarily exhibit ramping behavior. Spatial mapping of the order of activated neurons did not reveal any consistent associations or directional waves of activity in the transverse plane of view available (Figures 1E and 1F).

These observations suggest a high level of functional homogeneity among the ARN^{KISS} neuron population with, remarkably, almost all recorded cells contributing to SEs. However, this is achieved through heterogeneous patterns of individual neuron activation involving intermittent contributions to on-going SEs, variable ramping behavior, and flexible orders of activation.

Development of an acute brain slice preparation exhibiting spontaneous ARN^{KISS} neuron synchronizations

Acute brain slice studies were undertaken in a new mouse line in which *Kiss1*-Cre mice were crossed onto a Cre-dependent, Tet-amplified GCaMP6s mouse line (Ai162D). Histological assessment in coronal brain slices revealed the typical kisspeptin neuron pattern of cell body distribution for GCaMP in the ARN and rostral periventricular nuclei. Dual labeling with well-characterized kisspeptin antisera demonstrated that 98.6% ± 0.9% of kisspeptin neurons expressed GCaMP6, while 79.7% ± 3.3% of GCaMP-positive cells were immunoreactive for kisspeptin (n = 5) (Figure S2).

Taking insight from brain slice studies examining another central pattern generator,[19] we found that modifications to the brain slice thickness (320 μm), extracellular potassium ion (3.5 mM) and glucose (30 mM) concentrations alongside the use of Kiss1-Cre,Ai126D mice enabled spontaneous synchronized ARN^{KISS} neuron GCaMP activity to be visualized *in vitro* (Figure 2A). Coronal brain slices targeted at the middle and caudal aspects of the ARN were examined with up to 25 kisspeptin neurons clearly visualized in a plane of focus for simultaneous imaging. Abrupt increases in GCaMP signal were detected in an on-going manner in all active cells (Figure 2A). Episodes of activity were defined as events greater than two standard deviations of trace mean and events in two or more cells with peak amplitudes occurring within 10 s of each other were considered to represent periods of synchronization (Figure 2A). To differentiate these synchronizations from the population SEs recorded with photometry *in vivo*, these are termed "miniature" SEs (mSEs).

Dual GCaMP imaging whole-cell current-clamp recording of ARN^{KISS} neurons (n = 4 slices) revealed that calcium episodes were always associated with a short burst of firing (~8 s duration) during which frequencies approached 20 Hz at their peak (Figure 2B). Overall, whole-cell recordings from ARN^{KISS} neurons

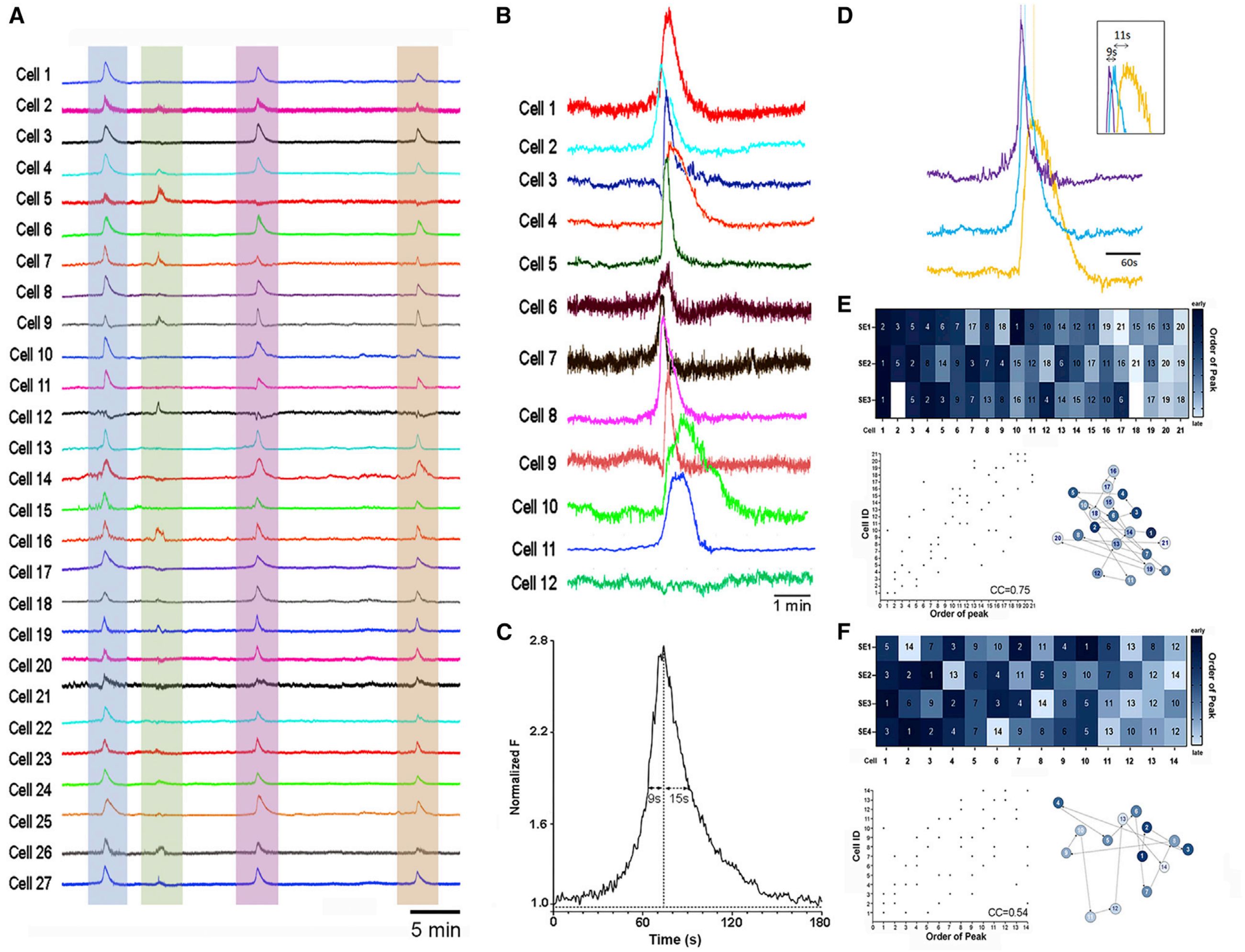

Figure 1. Synchronicity of individual ARN^{KISS} neurons *in vivo*

(A) Representative GCaMP6 traces from 27 individual ARN^{KISS} neurons recorded with a GRIN lens miniscope from a freely behaving GDX male mouse. Four synchronization events (colored) are observed with variable participation of individual neurons.

(B) Higher power images of GCaMP6 fluorescence levels from 12 selected ARN^{KISS} neurons over a single synchronization event.

(C) Average profile of GCaMP fluorescence in ARN^{KISS} neurons during a synchronization event.

(D) Representative example showing the differences in the timing of event peaks for the first, middle, and last ARN^{KISS} neurons in a synchronization event.

(E and F) Two representative examples showing the temporal relationships between individual ARN^{KISS} neurons (14 and 21 cells) across 3–4 synchronization events (SEs). The heatmap codes for order with early neurons in dark blue and late neurons in light blue. Empty squares indicate that the cell did not participate in the SE. A correlation scatterplot of the same information is provided below. Alongside is given the spatial location of individual ARN^{KISS} neurons in the transverse imaging plane showing their sequence of activity for the first SE.

in this preparation had a mean capacitance of 21.4 ± 1.25 pF, input resistance of 597 ± 21.1 MΩ, and resting membrane potential of −61.4 mV (n = 14 slices). Inclusion of tetrodotoxin (1 μM) in the aCSF completely abolished all calcium events and mSEs (n = 4 slices) (Figure 2C). This indicates that short bursts of synchronized firing among ARN^{KISS} neurons are responsible for mSEs in this brain slice preparation.

Characterization of spontaneous events and mSEs in brain slices

In calculating the rates of both calcium events in individual cells and mSEs, we controlled for the variation in the number of neurons recorded in each experiment by dividing by the number of cells recorded. This provides the mean rate at which each recorded neuron either exhibits a calcium event or takes part in an mSE.

In slices prepared from 36 intact males, calcium events occurred at a rate of 11.3 ± 1.0 events/cell/h and mSEs at 6.5 ± 0.9 mSEs/cell/h. The mean half-width of calcium events was 14.5 ± 1.2 s with a rise time of 4.3 ± 0.4 s and decay of 6.5 ± 0.2 s. Slices prepared from 25 GDX males exhibited slower rates of activity with 7.0 ± 0.7 events/cell/h (p = 0.0035; Mann-Whitney) and 3.5 ± 0.5 mSEs/cell/h (p = 0.035; Mann-Whitney). Compared with intact mice, the profile of individual events was not different in GDX mice with a mean half-width of 17.2 ± 1.7 s, raise time of 5.0 ± 0.7 s, and decay of 6.5 ± 0.5 s.

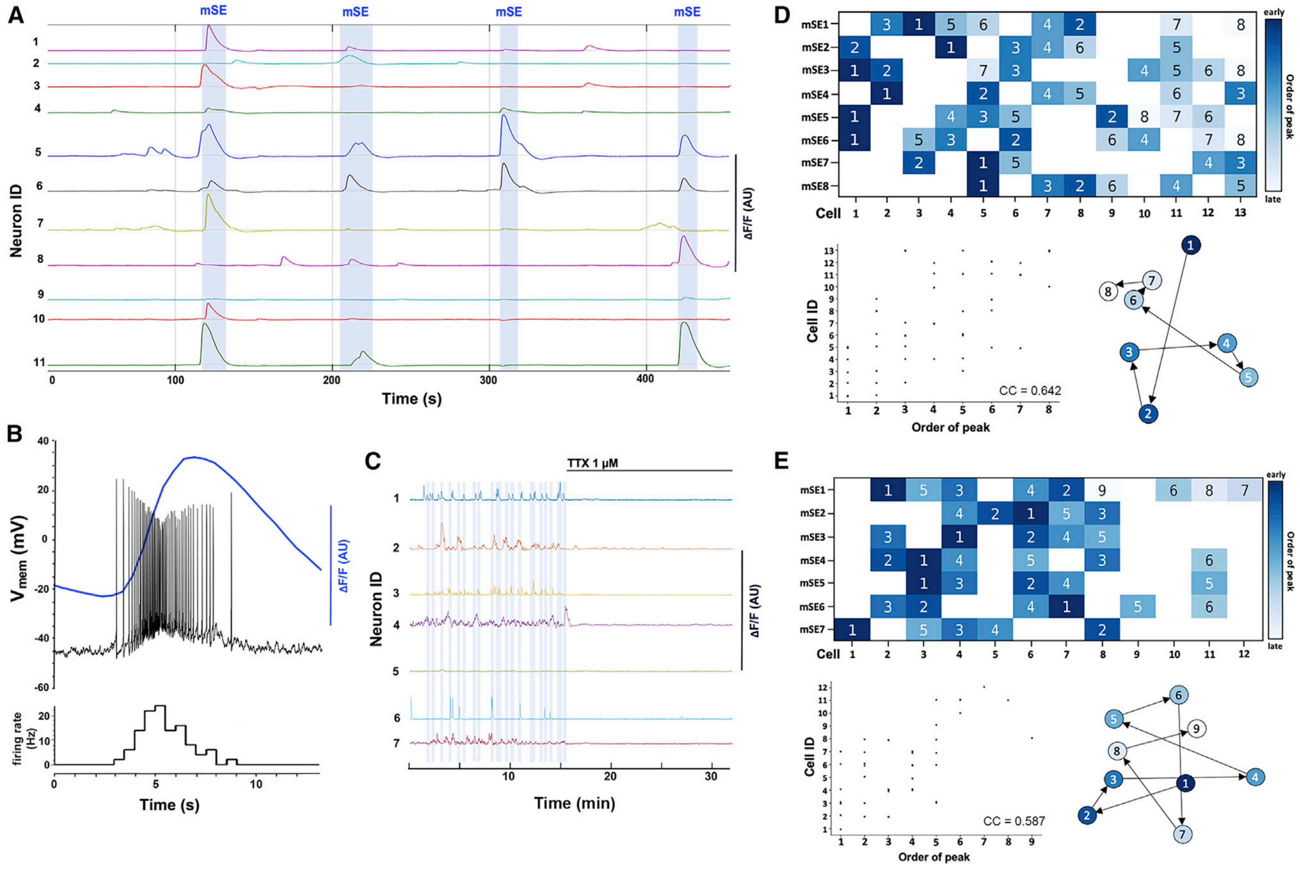

Figure 2. Spontaneous synchronous burst firing and GCaMP fluorescence in ARN^{KISS} neurons *in vitro*

(A) Traces showing GCaMP6 fluorescence levels recorded from 11 ARN^{KISS} neurons in the acute brain slice. Periods of synchrony termed miniature synchronization events (mSEs) are highlighted by blue shading.

(B) Dual GCaMP6-current-clamp whole-cell electrical recording from an ARN^{KISS} neuron over the time of an event. The histogram below shows firing rate (Hz) in 0.5 s bins.

(C) Long recording showing GCaMP fluorescence from seven ARN^{KISS} neurons in which TTX was added to the aCSF as indicated.

(D and E) Two representative examples showing the temporal relationships between individual ARN^{KISS} neurons (13 and 12 cells) across eight and seven miniature synchronization events (mSEs). The heatmap codes for order with early neurons in dark blue and late neurons in light blue. Empty squares indicate that the cell did not participate in the SE. A correlation scatterplot of the same information is provided below. Alongside is given the spatial location of individual ARN^{KISS} neurons in the coronal imaging plane showing their sequence of activity for the first SE.

All visible ARN^{KISS} neurons exhibited episodes of activity that were synchronized with at least one other ARN^{KISS} neuron.

The spatiotemporal participation of individual ARN^{KISS} neurons in synchronizations was analyzed from mSEs involving $\geq$5 cells (n = 7 slices with 3–9 mSEs). Although every neuron was involved in at least one mSE, their participation in on-going mSEs was inconsistent. No neurons were involved in every mSE while some cells were only active during a single mSE and, on average, a single neuron participated in 48.1% ± 2.2% of mSEs. Nevertheless, a moderate degree of consistency in the order of firing was apparent with the cells first activated during one mSE being more likely to be among the first cells activated on a subsequent mSE (Figures 2D and 2E). For example, Cell 1 in heatmap D was the first cell to fire in three of the four mSEs it participated in (out of a total of eight mSEs). In contrast, Cell 2 in heatmap E was activated first, second, fourth, and fifth in the five mSEs it participated in (out of seven mSEs) (Figures 2D and 2E). The mean Spearman correlation coefficient was 0.64 (range 0.46–0.91), with p values below the Bonferroni-corrected significance threshold (0.007) in every case, ranging from 0.002 to 0.0000001 (Figures 2D and 2E). The spatial order of activation was assessed in the same slices with no apparent anatomic or temporal relationship detected between cells in the experimental field of view (Figures 2D and 2E). These results show that while all ARN^{KISS} neurons can exhibit synchronous firing with one or more neurons, this occurs in a mostly unpredictable manner.

ARN^{KISS} neurons are only weakly coupled

The ability to identify individual ARN^{KISS} neurons involved in synchronous activity provided the opportunity to examine the strength of coupling between individual cells. Kisspeptin neurons exhibiting synchronous activity were identified and one cell in the group was selected for whole-cell patch clamp. The patched neuron was driven to fire at approximately 20 Hz

stimulation for 10–15 s by gradual 20–30 pA current injection and the effects on GCaMP fluorescence observed in all other ARN^{KISS} neurons in the slice. Patched cells exhibited spontaneous episodes of burst firing that were synchronized in a stochastic manner with increases in GCaMP signal in other neurons (Figures S3A and S3B). However, the activation of a patched neuron had no consistent effects on GCaMP fluorescence in other cells and, on average, only evoked a response in at least one other neuron in 31% of 89 trials (15 brain slices) (Figures S3A and S3B). Even when other cells were activated by the stimulated neuron, this was seldom repeatable (Figure S2B). As the activation of a single ARN^{KISS} neuron is usually insufficient, or at least unreliable, in initiating mSEs, it appears that individual ARN^{KISS} neurons are weakly coupled.

Critical role of AMPA-mediated transmission in ARN^{KISS} neuron synchronization *in vitro*

To explore the role of glutamate, NKB, and dynorphin in mediating mSEs, brain slices were prepared from intact and GDX male mice and appropriate receptor antagonists included into the aCSF for 20 min. Controls switching to aCSF alone resulted in no significant effects on the frequency of events (9.02 ± 1.72 events/cell/h pre-vehicle, 10.5 ± 1.76 events/cell/h during vehicle; n = 10 slices) or mSEs (Figure 3B).

Inclusion of a cocktail of the ionotropic glutamate receptor antagonists CNQX (20 μM) and D-AP5 (20 μM) resulted in an abrupt reduction in both calcium events and mSEs (Figure 3A). The event rate was reduced by 71% (13.7 ± 2.2 events/cell/h pre-drug and 3.9 ± 0.7 in CNQX + DAP5; p = 0.0078; Wilcoxon; n = 8) and mSE by 82% (8.7 ± 2.5 mSE/cell/h pre-drug, and 1.6 ± 0.4 in CNQX + DAP5; p = 0.0078; Wilcoxon) (Figure 3C). A very similar effect was observed in brain slices prepared from GDX mice with the event rate reduced by 58% (9.7 ± 1.0 events/cell/h pre-drug to 4.1 ± 1.0 drug-applied; p = 0.0078; Wilcoxon; n = 8) and mSEs by 78% (5.48 ± 0.5 mSEs/cell/h pre-drug to 1.2 ± 0.5 drug-applied; p = 0.023; Wilcoxon) (Figure 3E). To assess the contribution of AMPA receptors, slices from intact male mice were exposed to CNQX alone with an identical result observed (Figure 3D). Events per cell were reduced by 76% (17.8 ± 2.8 events/cell/h pre-drug to 6.1 ± 1.2 in CNQX; p = 0.031; Wilcoxon; N = 6) and mSEs by 75% (11.5 ± 1.6 pre-drug to 2.9 ± 0.7 in CNQX; p = 0.031; Wilcoxon) (Figure 3D). These observations demonstrate that glutamate signaling through the AMPA receptor is critical for ARN^{KISS} neuron synchronization in the brain slice.

No role for NKB or dynorphin transmission in ARN^{KISS} neuron synchronization *in vitro*

To explore the role of NKB in ARN^{KISS} neuron synchronization, the tachykinin receptor antagonists SDZ-NKT 343 (1 μM), GR94800 (1 μM), and SB 222200 (3 μM) targeting NK1R, NK2R, and NK3R, respectively, were bath applied as a cocktail. This combination has been shown previously to completely antagonize NKB signaling at ARN^{KISS} neurons.[14] Although variable, no consistent effect of the cocktail was found on events or mSEs in slices prepared from either intact or GDX males (Figures S4A–S4C). In intact males (n = 8), the event rate was 11.2 ± 2.2 events/cell/h pre-drug and 6.5 ± 0.8 during drug application (p = 0.15, Wilcoxon), while the mSE rate was 6.5 ± 1.8 pre-drug and 3.2 ± 0.7 in the presence of the cocktail (p = 0.25, Wilcoxon) (Figure S4B). In GDX males (N = 8), events occurred at 5.8 ± 1.2 events/cell/h pre-drug and 6.5 ± 1.9 drug-applied (p > 0.99, Wilcoxon) while mSEs occurred at 2.4 ± 0.9 mSEs/cell/h pre-drug and 4.3 ± 2.0 during cocktail application (p = 0.38, Wilcoxon) (Figure S4C). The effect of inhibiting tachykinin signaling on the profile of calcium increments was also investigated. No significant effects were identified on event half-width, rise time, or decay time in either intact or GDX males (Table S1).

The kappa opioid receptor antagonist norBNI (10 μM) was applied to inhibit dynorphin neurotransmission at ARN^{KISS} neurons.[14] In both intact males (n = 10) and GDX males (n = 9), application norBNI had variable effects but did not cause any significant change in the rates of events or mSEs (Figures S4D and S4E). The event rate was 11.8 ± 2.1 events/cell/h pre-drug and 7.5 ± 0.9 during norBNI (p = 0.084, Wilcoxon), while mSE rate was 7.2 ± 1.8 mSEs/cell/h pre-drug, and 3.9 ± 0.7 during norBNI (p = 0.28, Wilcoxon). In GDX males, events occurred at 5.7 ± 1.2 events/cell/h pre-drug, and 8.2 ± 1.8 during norBNI (p = 0.42, Wilcoxon), while mSEs occurred at 2.8 ± 0.8 mSEs/cell/h pre-drug and 5.0 ± 1.9 during norBNI (p = 0.82, Wilcoxon) (Figure S4F). No significant changes in half-width, rise time, or decay time were found in either intact or GDX males following treatment with norBNI (Table S1).

Miniature SEs are likely to occur *in vivo*

Short episodes of synchronized burst firing among kisspeptin neurons generate mSEs *in vitro* and emergent activity within this network is proposed to underlie the full SEs observed *in vivo*. To explore whether mSEs may be recordable *in vivo*, GCaMP fiber photometry recordings were undertaken from intact male mice for 5 h during which, on average, only a small number of SEs occur. In recordings with high amplitude SEs, presumably resulting from the optic fiber being very close to the kisspeptin population, numerous on-going low-amplitude events were observed amidst full SEs (Figure S5A). Defined by an amplitude that raises beyond one standard deviation of the mean and a duration ≥45 s, these low-amplitude events were found to have the same profile and dynamics of full SEs when normalized for amplitude (Figure S5B). Using these criteria, low-amplitude SEs occur at a rate of once every 19.5 ± 3.5 min in intact male mice (n = 5). It is likely that these low-amplitude events are the *in vivo* equivalents of mSEs recorded *in vitro*.

Roles of glutamate and neuropeptide transmission in ARN^{KISS} neuron synchronizations *in vivo*

The observations above indicate that glutamate transmission is essential for ARN^{KISS} neuron mSEs with little or no role for tonic tachykinin or dynorphin signaling in the acute brain slice. To assess the roles of these transmitters *in vivo*, the GCaMP fiber photometry was combined with a microinfusion approach to allow glutamate and neuropeptide transmission to be modified within the ARN while simultaneously recording SEs in freely behaving GDX male mice. Preliminary studies identified that the infusion of 1 μL of fluid over 10 min was compatible with reasonably stable photometry recordings. As such, an experimental strategy was adopted of infusing vehicle or receptor

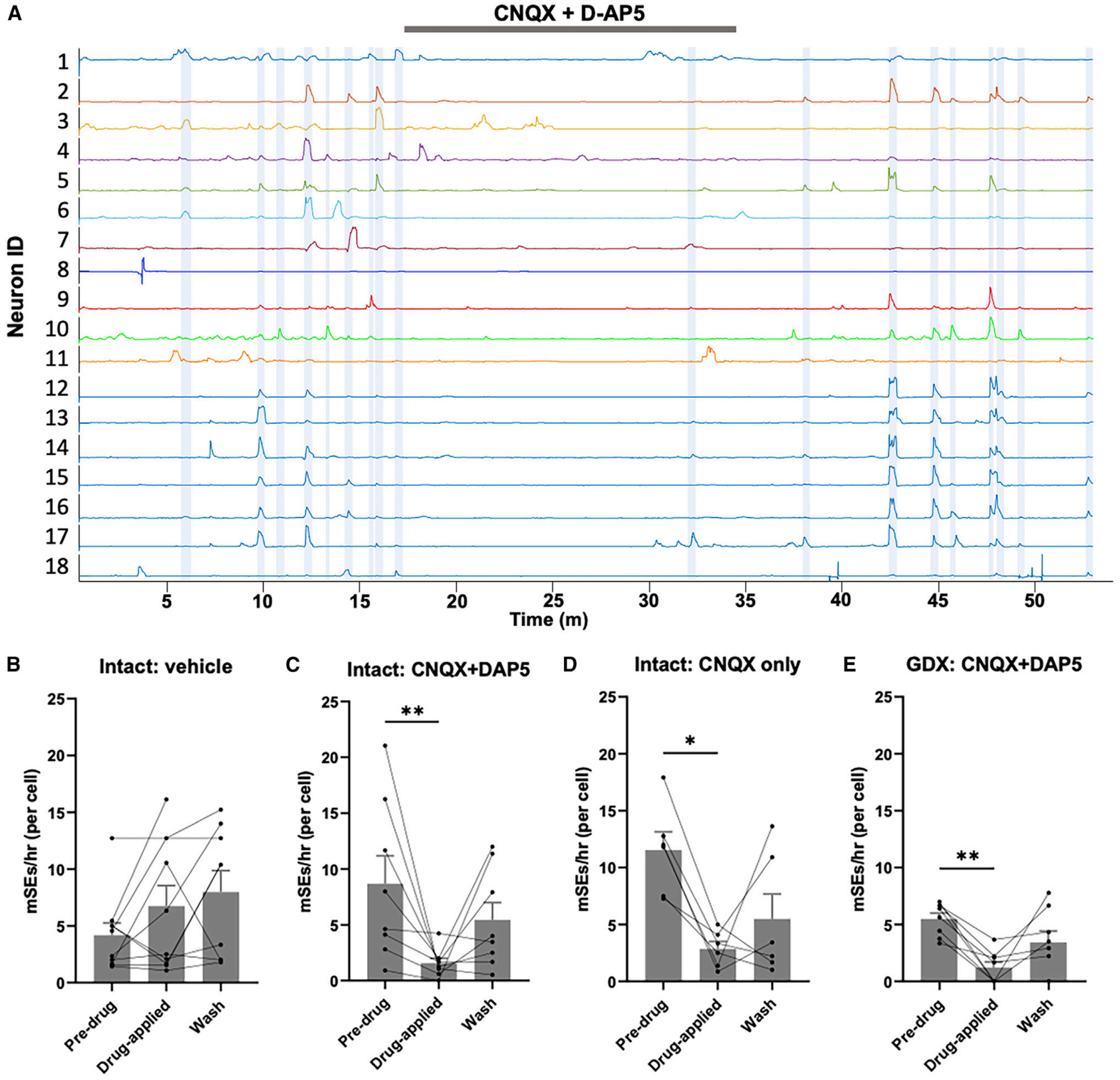

Figure 3. Glutamatergic signaling is essential for ARN^{KISS} neuron synchronization *in vitro*

(A) Example brain slice showing the effect of 15-min exposure to CNQX (20 μM) and D-AP5 (20 μM) on GCaMP fluorescence recorded from 18 neurons. mSEs are indicated by blue shading.

(B–E) Histograms showing the individual data points and mean (±SEM) mSE rate for the pre-drug, drug-applied and wash periods in response to vehicle, CNQX + AP5, CNQX alone in slices from intact male mice and the effect of CNQX and AP5 in GDX mice (*$p < 0.05$, **$p < 0.01$, Wilcoxon).

antagonists for 10 min after 50 min of a 120-min photometry recording session. The probability of an SE occurring and its amplitude during the infusion period was determined alongside the mean fluorescence level before, during, and after the infusion period. To test the efficacy of this approach, infusions of TTX (5 μM) were made and resulted in all SEs being abolished during the 10-min infusion period in four out of four trials with only a gradual recovery observed (n = 4) (Figure 4A).

Infusion of vehicle (n = 8) had no effect on the occurrence or amplitude of SEs. At least one SE was observed during the 10-min infusion period in seven of eight trials (87.5%) with no significant difference in GCaMP fluorescence before, during, or after infusion. No difference in SE amplitude was detected (1.1 normalized F). In contrast, after infusion of CNQX/AP5, both the probability of an SE occurring (33%; p = 0.03, chi-squared test) and mean fluorescence level (p = 0.03, one-way RM

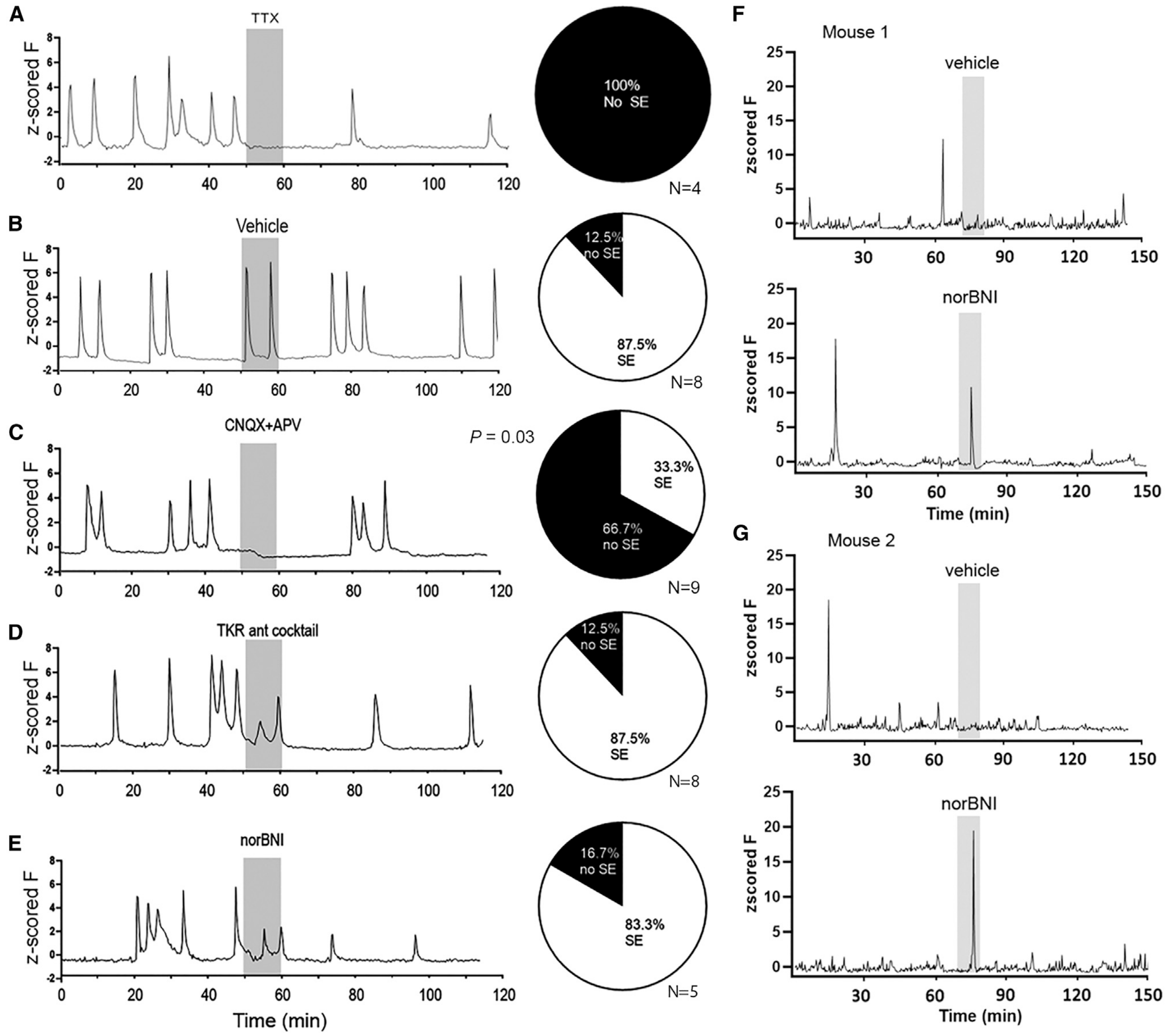

Figure 4. Effects of modulating intra-ARN transmission on ARN^{KISS} neuron synchronization in GDX and intact mice *in vivo*

(A–E) Left, representative GCaMP photometry traces showing the effects of 10-min intra-ARN infusions on SEs in GDX male mice. Middle, pie chart showing the chance of observing an SE during the 10-min infusion period.

(F and G) GCaMP photometry traces from two intact male mice given a 10-min intra-ARN infusion of vehicle or norBNI with at least a 1-week interval between infusions.

ANOVA followed by Dunnett's Multiple Comparison test) were significantly reduced (n = 9) (Figure 4C).

Neither the tachykinin receptor antagonist cocktail (GR94800, 10 μM; SDZ-NKT 343, 10 μM; SB222200, 30 μM) (n = 8) nor the norBNI (5 μM) (n = 5) infusion altered the probability of an SE occurring (87.5% and 83.3%, respectively) (Figures 4D and 4E) or mean GCaMP fluorescence level. However, the tachykinin receptor antagonist cocktail reduced the amplitude of individual SEs by 21% (p = 0.02, one-sample t test), while norBNI significantly reduced amplitude by 51% (p = 0.01, one-sample t test) (Figures 4D and 4E). To address whether norBNI impacts SE termination, the amplitude of SEs was normalized, and their half-width duration and decay time were compared before and during/after the infusion period. No parameters were significantly changed (half-width duration, 33.8 ± 9.0 versus 37.2 ± 9.7 s; decay time, 28.0 ± 2.9 versus 29.0 ± 2.3 s).

NKB modulates glutamate-evoked ARN^{KISS} neuron synchronizations

The *in vitro* and *in vivo* data demonstrate a critical role for glutamate transmission in enabling kisspeptin neurons to synchronize. Surprisingly, given the documented excitatory effects of NKB on ARN^{KISS} neurons,[14,15,18,20] the inhibition of tachykinin signaling had no effect on the initiation of SEs *in vitro* or *in vivo,*

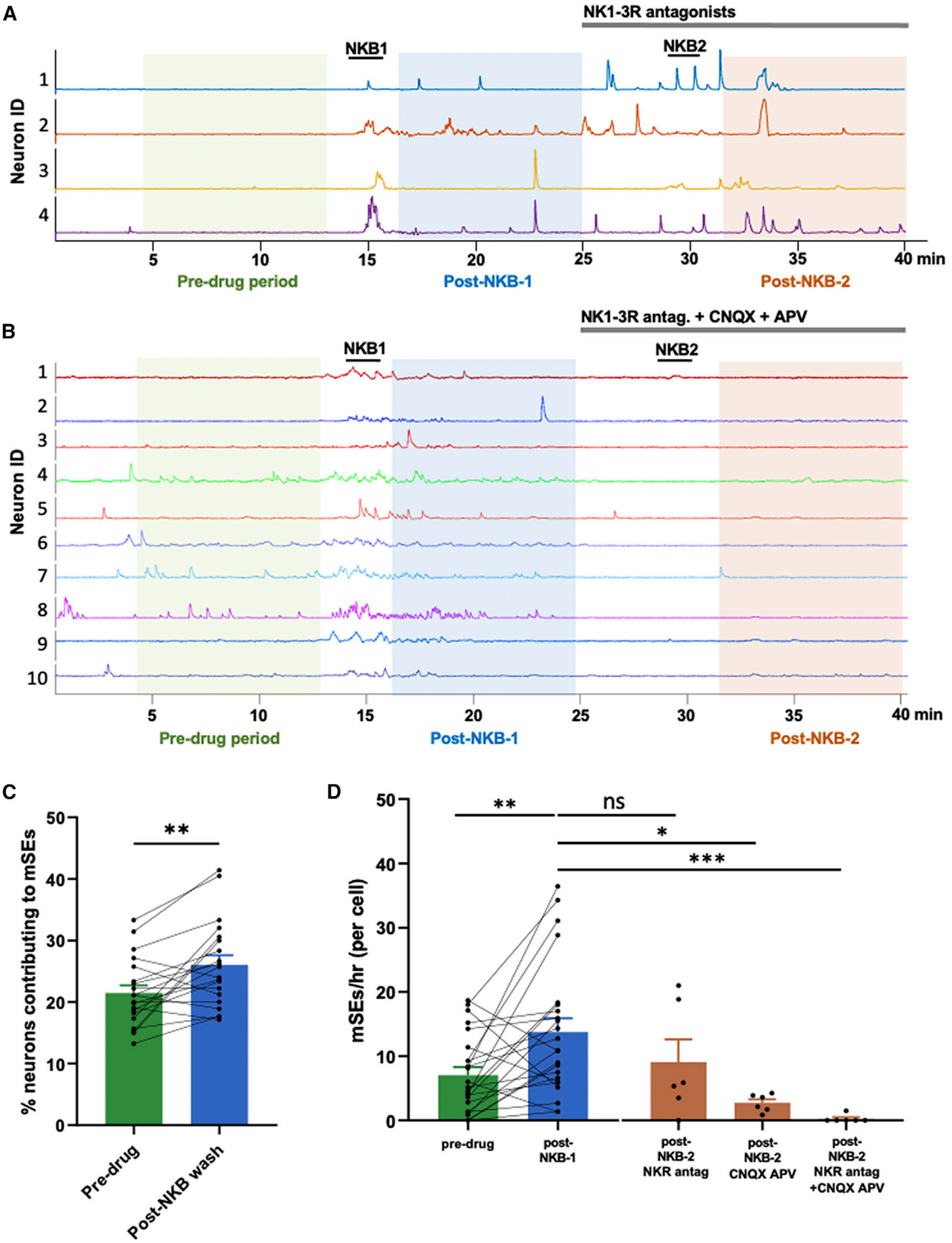

Figure 5. NKB modulates glutamate-driven ARN^KISS neuron synchronizations *in vitro*

(A) GCaMP traces from intact male mice with four neurons showing the acute stimulatory effect of NKB (NKB1; 50 nM) and generation of on-going mSEs in the "Post-NKB-1" period. This is followed by the addition of the tachykinin receptor (NK1-3R) antagonist cocktail and a second exposure to NKB (NKB2) with a second "Post-NKB-2" period. Each analysis period is shaded.

(B) GCaMP traces from 10 neurons receiving the same treatment as in (A), except for the addition of CNQX and AP5 with the tachykinin receptor antagonist cocktail.

(legend continued on next page)

but did result in a 20% reduction in SE amplitude *in vivo*. This suggested that NKB may act to facilitate synchronizations evoked by glutamate.

Using the brain slice preparation, we found that a short 90-s exposure to NKB resulted in an immediate increase in GCaMP signal followed by a prolonged period (post-NKB) of increased mSE frequency (7.12 ± 1.27 mSEs/cell/h pre-drug and 13.8 ± 2.13 during the post-NKB, Figures 5A and 5D) (n = 23 slices; p = 0.00049, Wilcoxon). The number of individual ARN^{KISS} neurons contributing to each mSE was significantly increased by NKB during this wash period (Figure 5C) from 21.5% to 26.0% (n = 20 slices; p = 0.013, Wilcoxon) (Figure 5C). To test the importance of glutamate and tachykinin signaling to these prolonged effects of NKB, a second NKB test (NKB2) and wash period (post-NKB2) was examined in the presence of the tachykinin 1–3 receptor antagonist cocktail (n = 6) or CNQX/APV (n = 6) alone, or both (n = 6). The NKB2 had no direct effect in the presence of the tachykinin receptor antagonist cocktail: even so, mSEs continued to be observed during the period following (Figures 5A and 5D). However, in contrast, there was an immediate suppression of mSEs in the presence of CNQX/APV alone (p = 0.029; Kruskal-Wallis) or the combined tachykinin cocktail and CNQX/AP5 (p = 0.0001; Kruskal-Wallis) (Figures 5B and 5D). Together, these results indicate that glutamatergic signaling is critical for NKB to potentiate synchronizations among ARN^{KISS} neurons.

Dynorphin controls the onset of SEs in a state-dependent manner *in vivo*

Given prior evidence for a potent suppressive effect of dynorphin on ARN^{KISS} neuron excitability,[14,15,18] it was curious to find no evidence for kappa opioid signaling in the duration or termination of an SE either *in vitro* or *in vivo*. This may have resulted from the brain slice having little on-going dynorphin release and the examination of GDX males *in vivo* where the pulse generator is uninhibited and considered "free-running" at its maximal state.

To test the state dependence of kappa opioid signaling *in vivo*, a further series of 10-min microinfusion-GCaMP photometry studies were undertaken but in intact male mice where SEs occur on average every 3 h.[8] As expected, synchronizations rarely occurred during the vehicle infusion period (Figures 4F and 4G) with only one of seven mice showing an SE during this 10-min interval. In contrast, a single SE occurred within 5–7 min of beginning the norBNI infusion in six of seven mice (Figures 4F and 4G). The norBNI-evoked SEs were similar in profile to those occurring spontaneously in each mouse, although the mean amplitude was smaller by 39% (two-way ANOVA followed by Sidak's multiple comparison test). Alongside data from GDX mice (Figure 4E), these observations demonstrate a marked state dependence in the role of dynorphin signaling to suppress the initiation of SEs *in vivo*.

Intrinsic mechanisms contributing to SE termination

The differing profiles of SE termination observed for individual neurons in the GRIN lens recordings (Figure 1D) suggested that intrinsic mechanisms may be of particular importance in SE termination. One possibility, given the well-characterized rapid desensitization of the AMPA receptor,[21] may be that the excitatory glutamate stimulus itself is short-lived and determines the duration of synchronization. To test this, we returned to the *in vitro* brain slice preparation and examined the effects of suppressing AMPA receptor desensitization with cyclothiazide[22] on mSEs. We found that application of 100 μM cyclothiazide dramatically increased mSE frequency (5.66 ± 1.29 mSEs/cell/h pre-drug and 17.1 ± 1.86 during drug application; n = 8 slices; p = 0.0078, Wilcoxon; Figures 6A and 6B), as well as event frequency (8.69 ± 1.33 events/cell/h pre-drug and 19.9 ± 1.89 during drug application; p = 0.0078, Wilcoxon). However, no significant changes were observed in the average mSE duration (Table S1) and cell-attached recordings of action currents (Figure 6A) revealed that burst firing remained during the inhibition of AMPA receptor desensitization. While confirming the key role of AMPA receptor signaling in ARN^{KISS} neuron synchronization, the data do not support a role for AMPA receptor desensitization in SE termination.

Another mechanism for SE termination could involve intrinsic activity-dependent inhibition through the big and small current calcium-activated potassium channels (BK and SK). These channels, activated by intracellular calcium entry, underlie spike frequency adaptation (SFA) that serves to slow firing after intense activation.[23] As the spontaneous burst firing exhibited by kisspeptin neurons displays SFA (Figure 2B), whole-cell current-clamp recordings were made from individual kisspeptin neurons in brain slices prepared from intact male mice to examine the combined effect of the highly selective antagonists for SK (apamin) and BK (iberiotoxin; IbTX) channels. Current injection (20 pA) generated clear SFA and this was significantly reduced by the apamin/IbTX cocktail (Figures 6C–6E) with the pre-drug SFA index being 0.47 ± 0.07 compared with 0.62 ± 0.047 in the presence of the antagonists (Figure 6E; n = 5; p = 0.021, paired t test). We also noted that the highly variable interspike interval (ISI) displayed by ARN^{KISS} neurons during activation (Figure 6F), attributed to A-type currents via Kv4.2 channels,[24] was almost completely inhibited by blockade of SK and BK channels (Figure 6G). These observations demonstrate that ARN^{KISS} neurons use intrinsic calcium-activated potassium channels to limit their excitability during periods of high firing.

DISCUSSION

Multiple different mechanisms ranging from the existence of distinct pacemaker cells through to emergent network activity have been implicated in the genesis of episodic activity in central

(C) Individual data points (n = 20 slices) and mean (±SEM) histograms showing the percentage of ARN^{KISS} neurons participating in mSEs during the pre-drug period and after the first NKB exposure (post-NKB-1). **p < 0.01, Wilcoxon test.

(D) Individual data points (n = 23 slices) and mean (±SEM) color-coded histograms showing the rate of mSEs in the pre-drug period and post-NKB-1 (NKB-1) periods (**p < 0.01, Wilcoxon test) and the rates of mSEs in the second post-NKB period (NKB-2) for slices treated with the tachykinin receptor antagonist cocktail alone (n = 6; not significant), CNQX/APV (n = 6; *p < 0.05, Kruskal-Wallis), or a combination of tachykinin and ionotropic glutamate receptor antagonists (n = 6; ***p < 0.001, Kruskal-Wallis).

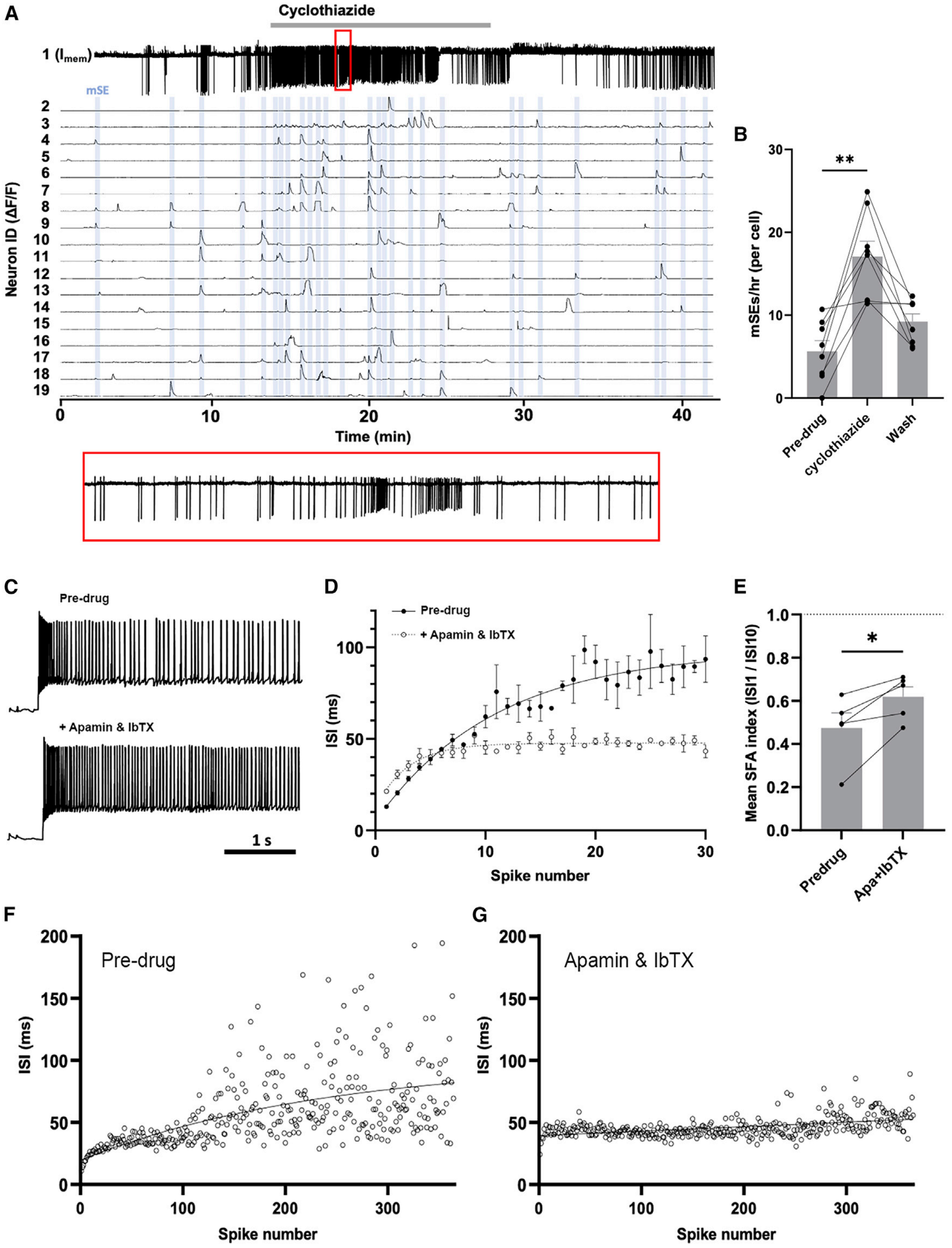

Figure 6. Intrinsic regulation of synchronization termination

(A) Representative brain slice recording showing the effect of 15-min exposure to cyclothiazide (100 μM) on both action potential firing in one neuron (neuron 1), and GCaMP fluorescence recorded from a further 18 neurons (neurons 2–19). mSEs are indicated by blue shading. A 60-s period of membrane current trace (red box) is expanded below, illustrating the maintenance of discreet periods of burst firing.

(B) Histogram showing the individual data points and mean (±SEM) mSE rate for the pre-drug, cyclothiazide-applied and wash periods in intact male mice (n = 8; $p < 0.01$, Wilcoxon test).

(legend continued on next page)

pattern generators.[19,25,26] We show here that, despite the kisspeptin pulse generator ultimately displaying a remarkable level of functional homogeneity, this is likely based upon dynamic cell assemblies in which individual SEs are seeded by near-random emergent activity within sub-populations of the network. We propose a "Glutamate two-transition" model for ARN^{KISS} neuron synchronization in which a baseline of near-stochastic, weakly coupled glutamatergic signaling between neurons moves through two transition states to achieve the network eruption required for pulse generation (Figure 7). The first transition involves the exponential recruitment of neurons based on mutual glutamatergic excitation to a threshold state where many neurons are synchronously activated. The ease with which the network can achieve this state is partly dependent upon intrinsic dynorphin tone within the circuit. The second transition involves the NKB potentiation of the glutamate-driven synchronization to reach its full explosive state (Figure 7).

We demonstrate a brain slice preparation in which essentially all ARN^{KISS} neurons exhibit spontaneous intermittent burst firing that variably merges to form episodes of synchronized activity. The individual ARN^{KISS} neurons contributing to each mSE are unpredictable and the reliability of experimentally activating one kisspeptin from another within a synchronous cluster is poor. Nevertheless, there is a modest correlation among the order of ARN^{KISS} neuron activity within mSEs indicating that some cells may, on average, be more tightly coupled than others. Together, this suggests that the network synchronizes through the formation of dynamic cell assemblies in which a stochastic element dictates the cells that initiate the synchronization. Modeling approaches show that weakly coupled networks of this type are well suited to facilitating emergent network activity and can generate robust synchronized outputs with variable or even stochastic timing.[27,28] This may explain the relatively unpredictable activation onsets of pulse generator activity *in vivo*.[8] It is interesting to note that the behavior of neonatal ARN^{KISS} neurons in primary culture is markedly different with extremely regular and frequent oscillations in which every kisspeptin neuron contributes to every synchronization.[29] Although synaptic coupling will be different in cell cultures, this raises the possibility that ARN^{KISS} neuron connectivity may alter during development.

The mSEs observed in brain slices are proposed to represent a basal level of intermittent synchronous behavior between ARN^{KISS} neurons that is only occasionally transitioned to a full SE. This is most pronounced in intact mice where, on average, mSEs occur every 6 min in the brain slice compared with an SE every 180 min *in vivo*. While this may, in part, reflect that the slice only ever contains a subpopulation of partially deafferented of ARN^{KISS} neurons, it nevertheless demonstrates that a relatively fast rhythm of spontaneous synchronizations exists within the network. Importantly, this behavior is also observed *in vivo* where low-amplitude mSE events with the same dynamic as full SEs occur with an interval of ~20 min and appear to be the counterparts of the *in vitro* mSEs. These mSEs do not trigger pulses of LH.[8] Precisely how the ARN^{KISS} neurons transition from mSEs to the full SEs observed *in vivo* is unknown. One possibility is that weak coupling within the network gradually builds through emergent self-excitation to reach a circuit consensus for an SE.

Interpretations regarding the order of activation of individual ARN^{KISS} neurons *in vivo* or *in vitro* must be tempered by the fact that we have recorded at random from a very small subpopulation of neurons in one plane of focus. Nevertheless, it is notable that a weak correlation for the order of activity existed among recorded neurons suggesting that there may be variable strengths of coupling among microcircuits of kisspeptin neurons. A recent study has reported a much stronger order correlation between ARN^{KISS} neurons during SEs in ovariectomized (OVX) mice.[30] This may be due to the interesting phenomenon of "pacing" whereby external stimuli related to the 5-min tail-tip bleeding drives the pulse generator to operate at an abnormally fast 5-min interval in OVX mice. While these GCaMP microendoscope data have value, it remains that the definition of the precise patterning of ARN^{KISS} neuron firing leading up to and during an SE will require the implementation of neuron-specific electrical recordings *in vivo*.

Glutamate is the key neurotransmitter enabling ARN^{KISS} neurons to synchronize. Ionotropic glutamate receptor signaling was required for neurons to exhibit burst firing and synchronous activity in the brain slice while, *in vivo*, direct infusion of glutamate antagonists into the ARN suppressed SE initiation. As CNQX replicated the effects of CNQX and AP5 combined and the inhibition of AMPA receptor desensitization greatly facilitated mSE rate, the AMPA receptor is very likely to be the primary mediator of glutamate transmission in the network. The ARN^{KISS} neurons express abundant AMPA receptors,[31] exhibit recurrent collateral innervation,[18,32] and have been shown to release glutamate on ARN^{KISS} and other neurons in the ARN.[18,33] As noted above, we propose that mutual glutamatergic excitation among dynamic assemblies of ARN^{KISS} neuron results in the exponential recruitment of neurons to achieve the SE. Heightened glutamate transmission within the circuit may explain why constant low-frequency optogenetic stimulation of ARN^{KISS} neurons can enhance LH pulse frequency.[34] Although there has long been interest in the role of glutamate in the mechanism of pulsatile hormone secretion,[35–37] methodological issues including the intraventricular administration of compounds have precluded any detailed interrogation. For example, administering CNQX and AP5 into the ventricular system suppresses pulsatile LH secretion but

(C) Example voltage trace in whole-cell configuration showing the response of an ARN^{KISS} neuron to 20-pA square wave current injection both pre-drug and during the application of apamin (150 nM) and iberiotoxin (IbTX; 100 nM).

(D) Change in mean (± SEM) interspike intervals (ISI) from the 1st to 30th spike recorded from a representative ARN^{KISS} neuron over three 20-pA stimulations, both pre-drug and in apamin + IbTX.

(E) An index of spike frequency adaptation (SFA) was generated by dividing the first ISI by the 10th ISI before and during exposure to apamin + IbTX ($n = 5$; $p < 0.05$, paired t test).

(F) Representative data showing the marked variability in ISI that occurs with progressive firing during the 20-pA stimulation of an ARN^{KISS} neuron and (G) the effects of treatment with apamin and IbTX.

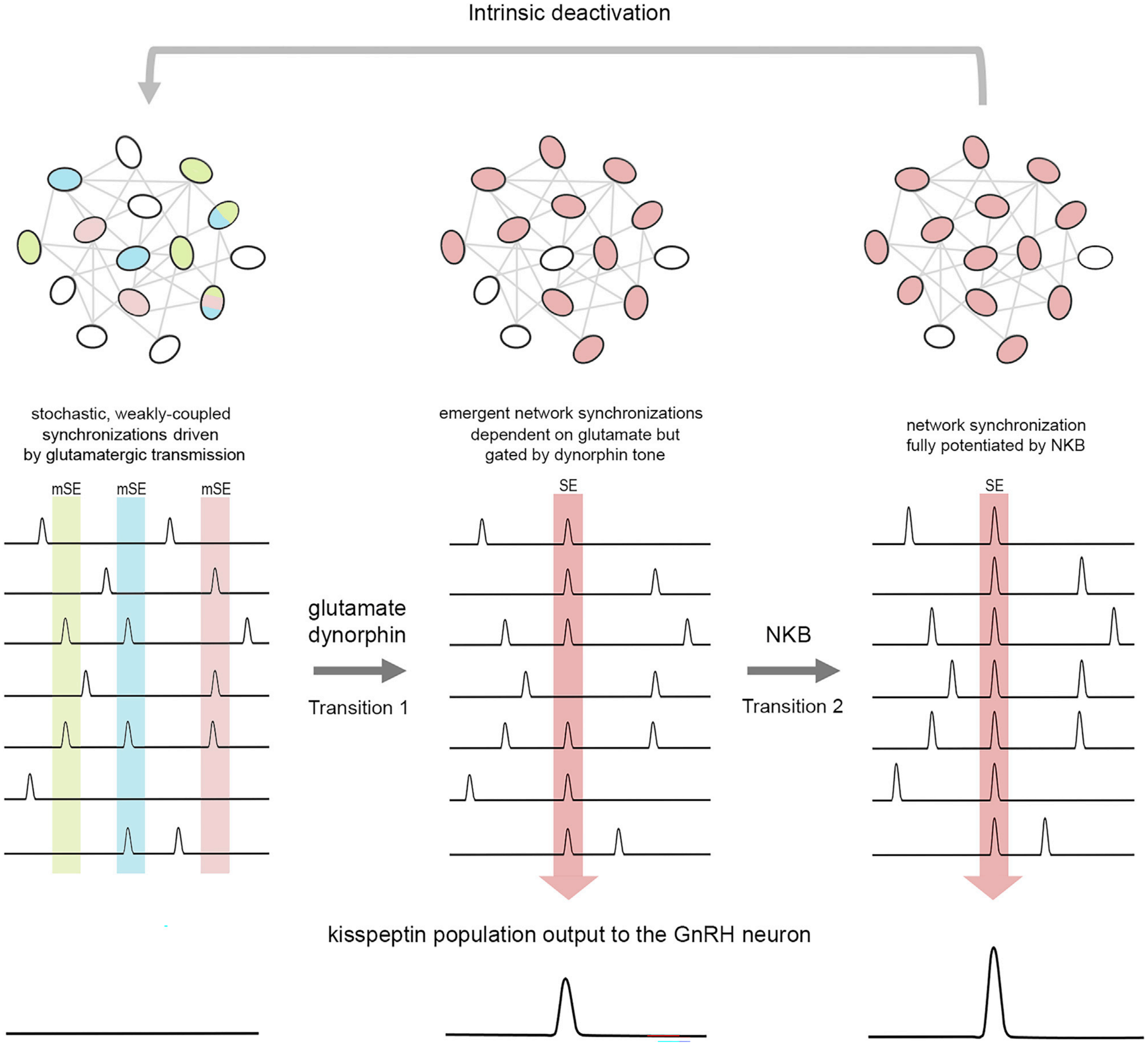

Figure 7. "Glutamate two-transition" model for ARN^{KISS} neuron synchronization
Stochastic, glutamate-dependent coupling between overlapping small groups of ARN^{KISS} neurons (different colors) generates on-going miniature synchronization episodes (mSEs). The first transition involves the emergence of a widespread population synchronization event (SE, pink) through exponential glutamate-driven self-excitation. This efficiency of this transition is modulated by dynorphin-kappa opioid tone within the network and also afferent inputs to the pulse generator that regulate its frequency of activity. The second transition involves the potentiation of already established synchronous activity by NKB increasing both the number of ARN^{KISS} neurons involved in an SE and their individual levels of excitation. Intrinsic mechanisms are then likely involved in synchronization termination.

the location of the neurons mediating those effects is unknown.[34,38] The observation here that infusion of CNQX/AP5 directly into the ARN suppresses SEs indicates that critical glutamatergic signaling is within the ARN. Whether this glutamate originates from ARN^{KISS} neurons themselves or a tonic extrinsic drive is unknown at present. However, the demonstration of glutamate-dependent synchronizations in the deafferented brain slice implies that the glutamate is of local origin within the ARN.

The transition of ARN^{KISS} neurons from mSEs to SEs is proposed to be a gated process that controls the frequency of the pulse generator (Figure 7). Given the wide number of afferent inputs proposed to control pulse generator frequency,[4,39] it is likely that several transmitters modulate the efficiency of this gate. We provide evidence here that dynorphin tone within the kisspeptin network is a key factor underlying the state dependence of gate operation. The local administration of norBNI into the ARN had no effect on SE interval in GDX mice but reliably initiated SEs

in intact mice. This demonstrates that dynorphin-kappa opioid transmission is suppressing the generation of SEs in intact mice, that exhibit slow pulse generator activity, but has no role in the free-running frequent SEs observed in GDX mice. The same situation may exist in rats where direct infusion of norBNI into the ARN of OVX animals has no effect on pulsatile LH secretion.[40] Interestingly, OVX ewes that nevertheless exhibit low-frequency LH pulses appear to maintain some dynorphin tone as intra-ARN norBNI enhances LH pulse frequency.[41] There is substantial experimental data demonstrating that dynorphin/opioid tone within the brain helps restrain LH pulse frequency under conditions of slow pulsatility but has no, or only a limited, role when the pulse generator is free-running.[10,42–45] Indeed, the administration of the opioid antagonist naloxone is a very robust strategy for increasing LH pulse frequency in the clinic.[46–48]

Precisely how dynorphin modulates ARN^{KISS} neurons remains unclear. Both pre- and post-synaptic mechanisms of dynorphin action have been reported for ARN^{KISS} neurons[14,15,18,31] with the presynaptic inhibition of AMPA transmission[18,31] being compatible with the proposed gating of SE initiation by dynorphin. However, it appears that dynorphin signaling plays multiple roles in this network. One counterintuitive observation made here is that the amplitude of SEs in GDX mice is suppressed by local infusion of norBNI into the ARN. The reason for this effect is unknown, but may result from high-frequency-evoked dynorphin release acting to limit the degree of ARN^{KISS} neuron activation during the SE.[18] If so, acutely removing this aspect of dynorphin signaling could result in hyperexcitable depolarization block at the peak of ARN^{KISS} neuron firing that would reduce intracellular calcium concentrations and, consequently, peak GCaMP signal.[49,50] The mechanisms and roles of pre- and post-synaptic regulation of ARN^{KISS} neuron excitability by dynorphin require further assessment.

The primary role of NKB is found to be that of potentiating synchronizations generated by glutamate transmission (Figure 7). Importantly, *in vivo*, tachykinin signaling was found to have no role in SE initiation but potentiated SE amplitude by approximately 20%. The intra-ARN infusion of a TAC3R antagonist similarly has no effect on LH pulse frequency in the OVX rat,[40] although the same approach in the OVX ewe slows LH pulse frequency.[41] Whereas glutamate will be released from ARN^{KISS} neurons terminals at very low firing frequencies, NKB transmission only occurs when the neuron reaches 10–20 Hz activation.[18] Thus, NKB appears to be acting as a classical frequency-dependent neuropeptide co-transmitter in potentiating the effects of amino acid transmission.[51] This is entirely compatible with brain slice studies showing that under conditions of high-frequency optogenetic activation, endogenous NKB can provide a slow depolarizing stimulus to ARN^{KISS} neurons.[18] This is also found to be partly dependent upon glutamate transmission in estrogen-treated OVX mice.[33] The release of NKB after the SE has commenced is likely to facilitate both the effectiveness and clustering of glutamate-driven synchronicity among the network (Figure 7). It is important to note that massive over-stimulation of the ARN^{KISS} neurons by exogenous NKB or Senktide results in the collapse of pulsatility in GDX animals.[10,40] likely due to depolarization block of the kisspeptin neuron or state-dependent desensitization of signaling within the GnRH-gonadotroph axis.[52]

A key prediction of a facilitatory role for NKB is that it would not be essential for pulse generation. Indeed, this may explain much of the observed experimental and clinical variability in relation to tachykinin signaling and the pulse generator.[48,53,54] Notably, the reversal of hypogonadotropic hypogonadism in individuals with tachykinin signaling mutations[55] suggests that NKB potentiation of the synchronization may be particularly important for the initiation of pulses at puberty but not thereafter.[54] We note that the proposed model is compatible with the presence of LH pulses in individuals with loss-of-function mutations in NKB that have, in addition, been treated with naloxone.[48]

No evidence was found for dynorphin playing a role in the termination of ARN^{KISS} neuron synchronizations *in vitro* or *in vivo*. We do, however, find that calcium-activated potassium channels operate within ARN^{KISS} neurons to limit their firing frequency at times of sustained activation. This suppression of intense ARN^{KISS} neuron firing would, in addition, be accompanied by reduced glutamate and NKB release. As such, these “intrinsic” mechanisms could contribute to SE termination (Figure 7), although it remains that other mechanisms may exist, and further investigation is warranted.

Limitations of the study

A key limitation of the current study and proposed “glutamate two-transition” model is that the work has been undertaken in male mice. Although it is thought that pulse generator operation is fundamentally the same in males and females,[4] it remains that future studies will need to ensure that the same mechanism operates in females. Equally, it will be important to evaluate whether the mechanism delineated here for mice is applicable to other species. Although there are rather few studies using the intra-ARN administration of receptor antagonists in relation to the pulse generator, results from the rat[40] are very similar to those reported here for the mouse, while data from sheep[41] differ in some respects.

In summary, we demonstrate here key features of the synchronization mechanism used by the central pattern generator controlling GnRH secretion in male mice. We propose a substantial departure from the existing “KNDy hypothesis” with glutamate as the primary transmitter generating synchronization within the network and neuropeptides as neuromodulators; dynorphin in state-dependent synchronization initiation and NKB operating to potentiate the magnitude of synchronizations (Figure 7). Kisspeptin itself does not contribute to the synchronization mechanism but is used as the exclusive output signal to the GnRH neuron.[56] It is expected that an accurate definition of GnRH pulse generator operation will be beneficial to the treatment of infertility in the clinic.

STAR★METHODS

Detailed methods are provided in the online version of this paper and include the following:

- KEY RESOURCES TABLE
- RESOURCE AVAILABILITY
  - Lead contact
  - Materials availability

- Data and code availability
- EXPERIMENTAL MODEL AND SUBJECT DETAILS
  - Animals
- METHOD DETAILS
  - Brain slice preparation
  - Brain slice calcium imaging and analysis
  - Brain slice electrophysiology and calcium imaging experiments
  - Stereotaxic surgery and injections
  - GCaMP6 GRIN lens miniscope imaging *in vivo*
  - GCaMP6 fiber photometry
  - Immunohistochemistry
  - Receptor and channel antagonists
- QUANTIFICATION AND STATISTICAL ANALYSES

SUPPLEMENTAL INFORMATION

Supplemental information can be found online at https://doi.org/10.1016/j.celrep.2022.111914.

ACKNOWLEDGMENTS

This work was supported by the Wellcome Trust (212242/Z/18/Z) and the New Zealand Health Research Council (16-148). J.-C.K. received a Doc.Mobility fellowship (P1ZHP1_184166) from the Swiss National Science Foundation. The authors thank Prof. Patrice Mollard, Montpellier and UCLA Miniscope team for assistance with the GRIN lens recordings. Dr. Karl Iremonger, University of Otago, New Zealand, and Prof. Bill Colledge and Dr. Sue Jones, University of Cambridge are thanked for comments on an early version of the manuscript. For the purpose of open access, the author has applied a Creative Commons Attribution (CC BY) license to any Author Accepted Manuscript version arising from this submission.

AUTHOR CONTRIBUTIONS

Conceptualization, A.E.H.; Methodology, S.Y.H. and P.G.M.; Investigation, S.Y.H., P.G.M., J.-C.K., S.G., M.P.N., S.H.Y., H.J.M.; Writing – Original Draft, A.E.H.; Writing – Review & Editing, S.Y.H., P.G.M., A.E.H.; Funding Acquisition, A.E.H.

DECLARATION OF INTERESTS

The authors declare no competing interests.

INCLUSION AND DIVERSITY

We support inclusive, diverse, and equitable conduct of research.

Received: August 2, 2022
Revised: October 31, 2022
Accepted: December 13, 2022

REFERENCES

1. Buzsáki, G., Logothetis, N., and Singer, W. (2013). Scaling brain size, keeping timing: evolutionary preservation of brain rhythms. Neuron *80*, 751–764. https://doi.org/10.1016/j.neuron.2013.10.002.
2. Ashhad, S., Kam, K., Del Negro, C.A., and Feldman, J.L. (2022). Breathing rhythm and pattern and their influence on emotion. Annu. Rev. Neurosci. *45*, 223–247. https://doi.org/10.1146/annurev-neuro-090121-014424.
3. Belchetz, P.E., Plant, T.M., Nakai, Y., Keogh, E.J., and Knobil, E. (1978). Hypophysial responses to continuous and intermittent delivery of hypopthalamic gonadotropin-releasing hormone. Science *202*, 631–633.
4. Herbison, A.E. (2018). The gonadotropin-releasing hormone pulse generator. Endocrinology *159*, 3723–3736. https://doi.org/10.1210/en.2018-00653.
5. Han, S.Y., McLennan, T., Czieselsky, K., and Herbison, A.E. (2015). Selective optogenetic activation of arcuate kisspeptin neurons generates pulsatile luteinizing hormone secretion. Proc. Natl. Acad. Sci. USA *112*, 13109–13114. https://doi.org/10.1073/pnas.1512243112.
6. Clarkson, J., Han, S.Y., Piet, R., McLennan, T., Kane, G.M., Ng, J., Porteous, R.W., Kim, J.S., Colledge, W.H., Iremonger, K.J., and Herbison, A.E. (2017). Definition of the hypothalamic GnRH pulse generator in mice. Proc. Natl. Acad. Sci. USA *114*, E10216–E10223. https://doi.org/10.1073/pnas.1713897114.
7. McQuillan, H.J., Han, S.Y., Cheong, I., and Herbison, A.E. (2019). GnRH pulse generator activity across the estrous cycle of female mice. Endocrinology *160*, 1480–1491. https://doi.org/10.1210/en.2019-00193.
8. Han, S.Y., Kane, G., Cheong, I., and Herbison, A.E. (2019). Characterization of GnRH pulse generator activity in male mice using GCaMP fiber photometry. Endocrinology *160*, 557–567. https://doi.org/10.1210/en.2018-01047.
9. O'Byrne, K.T., and Knobil, E. (1993). Electrophysiological approaches to gonadotrophin releasing hormone pulse generator activity in the rhesus monkey. Hum. Reprod. *8*, 37–40.
10. Wakabayashi, Y., Nakada, T., Murata, K., Ohkura, S., Mogi, K., Navarro, V.M., Clifton, D.K., Mori, Y., Tsukamura, H., Maeda, K.I., et al. (2010). Neurokinin B and dynorphin A in kisspeptin neurons of the arcuate nucleus participate in generation of periodic oscillation of neural activity driving pulsatile gonadotropin-releasing hormone secretion in the goat. J. Neurosci. *30*, 3124–3132. https://doi.org/10.1523/JNEUROSCI.5848-09.2010.
11. Plant, T.M. (2019). The neurobiological mechanism underlying hypothalamic GnRH pulse generation: the role of kisspeptin neurons in the arcuate nucleus. F1000Res. *8*, 982. https://doi.org/10.12688/f1000research.18356.1.
12. Skrapits, K., Borsay, B.Á., Herczeg, L., Ciofi, P., Liposits, Z., and Hrabovszky, E. (2015). Neuropeptide co-expression in hypothalamic kisspeptin neurons of laboratory animals and the human. Front. Neurosci. *9*, 29. https://doi.org/10.3389/fnins.2015.00029.
13. Goodman, R.L., Lehman, M.N., Smith, J.T., Coolen, L.M., de Oliveira, C.V.R., Jafarzadehshirazi, M.R., Pereira, A., Iqbal, J., Caraty, A., Ciofi, P., and Clarke, I.J. (2007). Kisspeptin neurons in the arcuate nucleus of the Ewe express both dynorphin A and neurokinin B. Endocrinology *148*, 5752–5760.
14. de Croft, S., Boehm, U., and Herbison, A.E. (2013). Neurokinin B activates arcuate kisspeptin neurons through multiple tachykinin receptors in the male mouse. Endocrinology *154*, 2750–2760. https://doi.org/10.1210/en.2013-1231.
15. Ruka, K.A., Burger, L.L., and Moenter, S.M. (2013). Regulation of arcuate neurons coexpressing kisspeptin, neurokinin B, and dynorphin by modulators of neurokinin 3 and kappa-opioid receptors in adult male mice. Endocrinology *154*, 2761–2771. https://doi.org/10.1210/en.2013-1268.
16. Navarro, V.M., Gottsch, M.L., Chavkin, C., Okamura, H., Clifton, D.K., and Steiner, R.A. (2009). Regulation of gonadotropin-releasing hormone secretion by kisspeptin/dynorphin/neurokinin B neurons in the arcuate nucleus of the mouse. J. Neurosci. *29*, 11859–11866. https://doi.org/10.1523/JNEUROSCI.1569-09.2009.
17. Lehman, M.N., Coolen, L.M., and Goodman, R.L. (2010). Minireview: kisspeptin/Neurokinin B/Dynorphin (KNDy) cells of the arcuate nucleus: a central node in the control of gonadotropin-releasing hormone secretion. Endocrinology *151*, 3479–3489. https://doi.org/10.1210/en.2010-0022.
18. Qiu, J., Nestor, C.C., Zhang, C., Padilla, S.L., Palmiter, R.D., Kelly, M.J., and Rønnekleiv, O.K. (2016). High-frequency stimulation-induced peptide release synchronizes arcuate kisspeptin neurons and excites GnRH neurons. Elife *5*, e16246. https://doi.org/10.7554/eLife.16246.
19. Ashhad, S., and Feldman, J.L. (2020). Emergent elements of inspiratory rhythmogenesis: network synchronization and synchrony propagation. Neuron *106*, 482–497.e4. https://doi.org/10.1016/j.neuron.2020.02.005.

CellPress
OPEN ACCESS

20. Hessler, S., Liu, X., and Herbison, A.E. (2020). Direct inhibition of arcuate kisspeptin neurones by neuropeptide Y in the male and female mouse. J. Neuroendocrinol. *32*, e12849. https://doi.org/10.1111/jne.12849.

21. Mosbacher, J., Schoepfer, R., Monyer, H., Burnashev, N., Seeburg, P.H., and Ruppersberg, J.P. (1994). A molecular determinant for submillisecond desensitization in glutamate receptors. Science *266*, 1059–1062. https://doi.org/10.1126/science.7973663.

22. Partin, K.M., Patneau, D.K., Winters, C.A., Mayer, M.L., and Buonanno, A. (1993). Selective modulation of desensitization at AMPA versus kainate receptors by cyclothiazide and concanavalin A. Neuron *11*, 1069–1082. https://doi.org/10.1016/0896-6273(93)90220-l.

23. Ha, G.E., and Cheong, E. (2017). Spike frequency adaptation in neurons of the central nervous system. Exp. Neurobiol. *26*, 179–185. https://doi.org/10.5607/en.2017.26.4.179.

24. Mendonça, P.R.F., Kyle, V., Yeo, S.H., Colledge, W.H., and Robinson, H.P.C. (2018). Kv4.2 channel activity controls intrinsic firing dynamics of arcuate kisspeptin neurons. J. Physiol. *596*, 885–899. https://doi.org/10.1113/JP274474.

25. Kiehn, O. (2016). Decoding the organization of spinal circuits that control locomotion. Nat. Rev. Neurosci. *17*, 224–238. https://doi.org/10.1038/nrn.2016.9.

26. Ramirez, J.M., and Baertsch, N.A. (2018). The dynamic basis of respiratory rhythm generation: one breath at a time. Annu. Rev. Neurosci. *41*, 475–499. https://doi.org/10.1146/annurev-neuro-080317-061756.

27. Carroll, M.S., and Ramirez, J.M. (2013). Cycle-by-cycle assembly of respiratory network activity is dynamic and stochastic. J. Neurophysiol. *109*, 296–305. https://doi.org/10.1152/jn.00830.2011.

28. Wang, X.J. (2010). Neurophysiological and computational principles of cortical rhythms in cognition. Physiol. Rev. *90*, 1195–1268. https://doi.org/10.1152/physrev.00035.2008.

29. Kim, D., Jang, S., Kim, J., Park, I., Ku, K., Choi, M., Lee, S., Heo, W.D., Son, G.H., Choe, H.K., and Kim, K. (2020). Kisspeptin neuron-specific and self-sustained calcium oscillation in the hypothalamic arcuate nucleus of neonatal mice: regulatory factors of its synchronization. Neuroendocrinology *110*, 1010–1027. https://doi.org/10.1159/000505922.

30. Moore, A.M., Coolen, L.M., and Lehman, M.N. (2022). In vivo imaging of the GnRH pulse generator reveals a temporal order of neuronal activation and synchronization during each pulse. Proc. Natl. Acad. Sci. USA *119*, e2117767119. https://doi.org/10.1073/pnas.2117767119.

31. Wang, L., Burger, L.L., Greenwald-Yarnell, M.L., Myers, M.G., Jr., and Moenter, S.M. (2018). Glutamatergic transmission to hypothalamic kisspeptin neurons is differentially regulated by estradiol through estrogen receptor alpha in adult female mice. J. Neurosci. *38*, 1061–1072. https://doi.org/10.1523/JNEUROSCI.2428-17.2017.

32. Yip, S.H., Boehm, U., Herbison, A.E., and Campbell, R.E. (2015). Conditional viral tract tracing delineates the projections of the distinct kisspeptin neuron populations to GnRH neurons in the mouse. Endocrinology *156*, 2582–2594. https://doi.org/10.1210/en.2015-1131.

33. Qiu, J., Rivera, H.M., Bosch, M.A., Padilla, S.L., Stincic, T.L., Palmiter, R.D., Kelly, M.J., and Rønnekleiv, O.K. (2018). Estrogenic-dependent glutamatergic neurotransmission from kisspeptin neurons governs feeding circuits in females. Elife *7*, e35656. https://doi.org/10.7554/eLife.35656.

34. Voliotis, M., Li, X.F., De Burgh, R.A., Lass, G., Ivanova, D., McIntyre, C., O'Byrne, K., and Tsaneva-Atanasova, K. (2021). Modulation of pulsatile GnRH dynamics across the ovarian cycle via changes in the network excitability and basal activity of the arcuate kisspeptin network. Elife *10*, e71252. https://doi.org/10.7554/eLife.71252.

35. Herbison, A.E. (2015). Physiology of the adult GnRH neuronal network. In Knobil and Neill's Physiology of Reproduction, T.M. Plant and A.J. Zeleznik, eds. (Academic Press), pp. 399–467.

36. Terasawa, E. (2001). Luteinizing hormone-releasing hormone (LHRH) neurons: mechanism of pulsatile LHRH release. Vitam. Horm. *63*, 91–129.

37. Brann, D.W., and Mahesh, V.B. (1997). Excitatory amino acids: evidence for a role in the control of reproduction and anterior pituitary hormone secretion. Endocr. Rev. *18*, 678–700.

38. Ping, L., Mahesh, V.B., and Brann, D.W. (1994). A physiological role for N-methyl-D-aspartic acid and non-N-methyl-D-aspartic acid receptors in pulsatile gonadotropin secretion in the adult female rat. Endocrinology *135*, 113–118.

39. Yeo, S.H., and Colledge, W.H. (2018). The role of Kiss1 neurons as integrators of endocrine, metabolic, and environmental factors in the hypothalamic-pituitary-gonadal Axis. Front. Endocrinol. *9*, 188. https://doi.org/10.3389/fendo.2018.00188.

40. Grachev, P., Li, X.F., Kinsey-Jones, J.S., di Domenico, A.L., Millar, R.P., Lightman, S.L., and O'Byrne, K.T. (2012). Suppression of the GnRH pulse generator by neurokinin B involves a kappa-opioid receptor-dependent mechanism. Endocrinology *153*, 4894–4904. https://doi.org/10.1210/en.2012-1574.

41. Goodman, R.L., Hileman, S.M., Nestor, C.C., Porter, K.L., Connors, J.M., Hardy, S.L., Millar, R.P., Cernea, M., Coolen, L.M., and Lehman, M.N. (2013). Kisspeptin, neurokinin B, and dynorphin act in the arcuate nucleus to control activity of the GnRH pulse generator in ewes. Endocrinology *154*, 4259–4269. https://doi.org/10.1210/en.2013-1331.

42. Whisnant, C.S., and Goodman, R.L. (1988). Effects of an opioid antagonist on pulsatile luteinizing hormone secretion in the Ewe vary with changes in steroid negative feedback. Biol. Reprod. *39*, 1032–1038.

43. Mostari, P., Ieda, N., Deura, C., Minabe, S., Yamada, S., Uenoyama, Y., Maeda, K.i., and Tsukamura, H. (2013). dynorphin-kappa opioid receptor signaling partly mediates estrogen negative feedback effect on LH pulses in female rats. J. Reprod. Dev. *59*, 266–272. https://doi.org/10.1262/jrd.2012-193.

44. Melis, G.B., Paoletti, A.M., Gambacciani, M., Mais, V., and Fioretti, P. (1984). Evidence that estrogens inhibit LH secretion through opioids in postmenopausal women using naloxone. Neuroendocrinology *39*, 60–63. https://doi.org/10.1159/000123956.

45. Bhanot, R., and Wilkinson, M. (1984). The inhibitory effect of opiates on gonadotrophin secretion is dependent upon gonadal steroids. J. Endocrinol. *102*, 133–141. https://doi.org/10.1677/joe.0.1020133.

46. Perkins, R.B., Hall, J.E., and Martin, K.A. (1999). Neuroendocrine abnormalities in hypothalamic amenorrhea: spectrum, stability, and response to neurotransmitter modulation. J. Clin. Endocrinol. Metab. *84*, 1905–1911. https://doi.org/10.1210/jcem.84.6.5823.

47. Tenhola, H., Sinclair, D., Alho, H., and Lahti, T. (2012). Effect of opioid antagonists on sex hormone secretion. J. Endocrinol. Invest. *35*, 227–230. https://doi.org/10.3275/8181.

48. Lippincott, M.F., León, S., Chan, Y.M., Fergani, C., Talbi, R., Farooqi, I.S., Jones, C.M., Arlt, W., Stewart, S.E., Cole, T.R., et al. (2019). Hypothalamic reproductive endocrine pulse generator activity independent of neurokinin B and dynorphin signaling. J. Clin. Endocrinol. Metab. *104*, 4304–4318. https://doi.org/10.1210/jc.2019-00146.

49. Peterson, B.Z., DeMaria, C.D., Adelman, J.P., and Yue, D.T. (1999). Calmodulin is the Ca2+ sensor for Ca2+ -dependent inactivation of L-type calcium channels. Neuron *22*, 549–558. https://doi.org/10.1016/s0896-6273(00)80709-6.

50. Dougalis, A.G., Matthews, G.A.C., Liss, B., and Ungless, M.A. (2017). Ionic currents influencing spontaneous firing and pacemaker frequency in dopamine neurons of the ventrolateral periaqueductal gray and dorsal raphe nucleus (vlPAG/DRN): a voltage-clamp and computational modelling study. J. Comput. Neurosci. *42*, 275–305. https://doi.org/10.1007/s10827-017-0641-0.

51. van den Pol, A.N. (2012). Neuropeptide transmission in brain circuits. Neuron *76*, 98–115. https://doi.org/10.1016/j.neuron.2012.09.014.

52. Han, S.Y., Cheong, I., McLennan, T., and Herbison, A.E. (2020). Neural determinants of pulsatile luteinizing hormone secretion in male mice. Endocrinology *161*, bqz045. https://doi.org/10.1210/endocr/bqz045.

53. Talbi, R., Ferrari, K., Choi, J.H., Gerutshang, A., McCarthy, E.A., Dischino, D., León, S., and Navarro, V.M. (2021). Characterization of the action of tachykinin signaling on pulsatile LH secretion in male mice. Endocrinology *162*, bqab074. https://doi.org/10.1210/endocr/bqab074.

54. True, C., Nasrin Alam, S., Cox, K., Chan, Y.M., and Seminara, S.B. (2015). Neurokinin B is critical for normal timing of sexual maturation but dispensable for adult reproductive function in female mice. Endocrinology *156*, 1386–1397. https://doi.org/10.1210/en.2014-1862.

55. Gianetti, E., Tusset, C., Noel, S.D., Au, M.G., Dwyer, A.A., Hughes, V.A., Abreu, A.P., Carroll, J., Trarbach, E., Silveira, L.F.G., et al. (2010). TAC3/TACR3 mutations reveal preferential activation of gonadotropin-releasing hormone release by neurokinin B in neonatal life followed by reversal in adulthood. J. Clin. Endocrinol. Metab. *95*, 2857–2867. https://doi.org/10.1210/jc.2009-2320.

56. Liu, X., Yeo, S.H., McQuillan, H.J., Herde, M.K., Hessler, S., Cheong, I., Porteous, R., and Herbison, A.E. (2021). Highly redundant neuropeptide volume co-transmission underlying episodic activation of the GnRH neuron dendron. Elife *10*, e62455. https://doi.org/10.7554/eLife.62455.

57. Franceschini, I., Yeo, S.H., Beltramo, M., Desroziers, E., Okamura, H., Herbison, A.E., and Caraty, A. (2013). Immunohistochemical evidence for the presence of various kisspeptin isoforms in the Mammalian brain. J. Neuroendocrinol. *25*, 839–851. https://doi.org/10.1111/jne.12069.

58. Yeo, S.H., Kyle, V., Morris, P.G., Jackman, S., Sinnett-Smith, L.C., Schacker, M., Chen, C., and Colledge, W.H. (2016). Visualisation of Kiss1 neurone distribution using a Kiss1-CRE transgenic mouse. J. Neuroendocrinol. *28*. https://doi.org/10.1111/jne.12435.

59. Schneider, C.A., Rasband, W.S., and Eliceiri, K.W. (2012). NIH Image to ImageJ: 25 years of image analysis. Nat. Methods *9*, 671–675. https://doi.org/10.1038/nmeth.2089.

60. Cai, D.J., Aharoni, D., Shuman, T., Shobe, J., Biane, J., Song, W., Wei, B., Veshkini, M., La-Vu, M., Lou, J., et al. (2016). A shared neural ensemble links distinct contextual memories encoded close in time. Nature *534*, 115–118. https://doi.org/10.1038/nature17955.

61. Daigle, T.L., Madisen, L., Hage, T.A., Valley, M.T., Knoblich, U., Larsen, R.S., Takeno, M.M., Huang, L., Gu, H., Larsen, R., et al. (2018). A suite of transgenic driver and reporter mouse lines with enhanced brain-cell-type targeting and functionality. Cell *174*, 465–480.e22. https://doi.org/10.1016/j.cell.2018.06.035.

62. Lerner, T.N., Shilyansky, C., Davidson, T.J., Evans, K.E., Beier, K.T., Zalocusky, K.A., Crow, A.K., Malenka, R.C., Luo, L., Tomer, R., and Deisseroth, K. (2015). Intact-brain analyses reveal distinct information carried by SNc dopamine subcircuits. Cell *162*, 635–647. https://doi.org/10.1016/j.cell.2015.07.014.

CellPress
OPEN ACCESS

STAR★METHODS

KEY RESOURCES TABLE

REAGENT or RESOURCE	SOURCE	IDENTIFIER
**Antibodies**		
Chicken anti-green fluorescent protein, GFP-1010	Aves Labs	RRID: AB_2307313
Sheep anti-Kisspeptin 52, AC053	(Franceschini et al.)[57]	N/A
Goat anti-chicken IgY (H + L) Alexa 488-conjugate, A-11039	Thermo Fisher Scientific	RRID: AB_2534096
Donkey anti-sheep F(ab')$_2$ fragment IgG (H + L) biotin-conjugate, 713-066-147	Jackson Immunoresearch	RRID: AB_2340717
Streptavidin Alexa 568-conjugate, S11226	Thermo Fisher Scientific	RRID: AB_2336132
Goat anti-chicken IgY (H + L) Alexa 488-conjugate, A-11039	Thermo Fisher Scientific	RRID: AB_2534096
Donkey anti-sheep F(ab')$_2$ fragment IgG (H + L) biotin-conjugate, 713-066-147	Jackson Immunoresearch	RRID: AB_2340717
Streptavidin Alexa 568-conjugate, S11226	Thermo Fisher Scientific	RRID: AB_2336132
**Bacterial and virus strains**		
AAV9-CAG-FLEX-GCaMP6s-WPRE-SV40	Addgene	#100842-AAV9<br>RRID:Addgene_100842
**Chemicals, peptides, and recombinant proteins**		
norBNI	Sigma-Aldrich UK	Cat#N1771
CNQX	Sigma-Aldrich UK	Cat#C127
D-AP5	Sigma-Aldrich UK	Cat#A8054
NKB	Sigma-Aldrich UK	Cat#N4143
SDZ-NKT 343	Tocris (Bio-Techne) UK	Cat#2394
GR 94800	Tocris (Bio-Techne) UK	Cat#1667
SB 222200	Tocris (Bio-Techne) UK	Cat#1393
Apamin	Tocris (Bio-Techne) UK	Cat#1652
Iberiotoxin	Tocris (Bio-Techne) UK	Cat#1086
Cyclothiazide	Tocris (Bio-Techne) UK	Cat#0713
TTX	Tocris (Bio-Techne) UK	Cat#1078
**Experimental models: Organisms/strains**		
Mouse:129-Kiss1$^{tm2(cre/GFP)Coll}$/H	(Yeo et al.)[58]	N/A
Mouse: B6.Cg-*Igs7*$^{tm162.1(tetO-GCaMP6s,CAG-tTA2)Hze}$/J	The Jackson Laboratory	RRID:IMSR_JAX:031562
**Software and algorithms**		
ImageJ	(Schneider et al.)[59]	https://imagej.nih.gov/ij/<br>RRID: SCR_003070
Open-source Neuromouse fiber photometry data acquisition system	Tussock Innovation and Argotech, New Zealand	https://www.otago.ac.nz/neuroendocrinology/resources/argotech.html
MiniscopeAnalysis algorithms	Sylvain Williams Lab, McGill University	https://github.com/etterguillaume/MiniscopeAnalysis
MATLAB (R2019a)	MathWorks	http://www.mathworks.com/products/matlab/<br>RRID:SCR_001622
Open-source Miniscope Data Acquisition Software	(Cai et al.)[60]	https://github.com/daharoni/Miniscope_DAQ_Software
LAS X (Leica Applications Suite) confocal microscopy software	Leica Microsystems	https://www.leica-microsystems.com/<br>RRID: SCR_013673

(*Continued on next page*)

Continued		
REAGENT or RESOURCE	SOURCE	IDENTIFIER
Other		
UCLA Open-source Miniscope	(Cai et al.)[60]	https://github.com/Aharoni-Lab/Miniscope-v4)
Multiple Fluid Injections Fiberoptic Cannulas	Doric Lenses, QC, Canada	https://neuro.doriclenses.com/
In vitro mSE analysis code	Zenodo repository	https://doi.org/10.5281/zenodo.7334481

RESOURCE AVAILABILITY

Lead contact

Further information and requests for resources and reagents should be directed to and will be fulfilled by the lead contact, Allan Herbison (aeh36@cam.ac.uk).

Materials availability

This study did not generate new unique reagents.

Data and code availability

Data reported in this paper will be shared by the lead contact upon request.

All original code has been deposited at Zenodo and is available as of the date of publication. DOIs are listed in the key resources table.

Any additional information required to reanalyze the data reported in this paper is available from the lead contact upon request.

EXPERIMENTAL MODEL AND SUBJECT DETAILS

Animals

Male 129S6Sv/Ev C57BL/6 *Kiss1*$^{Cre/+}$mice[58] alone or crossed on to the Ai162 (TIT2L-GC6s-ICL-tTA2)-D Cre-dependent GCaMP6s line (JAX stock #031562)[61] were group-housed in individually-ventilated cages with environmental enrichment under conditions of controlled temperature (22 ± 2°C) and lighting (12-h light/12-h dark cycle; lights on at 6:00h and off at 18:00h) with *ad libitum* access to food (RM1-P, SDS, UK) and water. All animal experimental protocols were approved by the University of Otago, New Zealand (96/2017) and the University of Cambridge, UK (P174441DE). Where required, mice were bilaterally gonadectomized under Isoflurane anesthesia at least 3 weeks prior to experimentation. Mice were 8–26 weeks old at the time of experimentation.

METHOD DETAILS

Brain slice preparation

Kiss1-Cre,Ai126D mice were anesthetized using isoflurane, decapitated and the brain removed into oxygenated, ice-cold slicing solution composed of (mM): NaCl 52.5; sucrose 100; glucose 25; $NaHCO_3$ 25; KCl 2.5; $CaCl_2$ 1; $MgCl_2$ 5; NaH_2PO_4 1.25; kynurenic acid 0.1 (95% O_2/5% CO_2). Coronal slices containing the ARN were prepared at 320 μm thickness using a Leica VT1200S tissue slicer (Leica Biosystems UK) before being transferred to a submersion chamber containing an oxygenated (95% O_2/5% CO_2) aCSF recording solution composed of (mM): NaCl 124; glucose 30; $NaHCO_3$ 25; KCl 3.5; $CaCl_2$ 1.5; $MgCl_2$ 1; NaH_2PO_4 0.5 and incubated at 30°C for 1–5 h prior to use.

Brain slice calcium imaging and analysis

Brain slices containing the ARN were transferred to the stage of an Olympus BX51WI upright microscope with differential interference contrast optics, and constantly perfused with oxygenated aCSF at 30 ± 1°C. The variation in intracellular calcium concentration ($[Ca^{2+}]_i$) of ARN^{KISS} neurons was estimated by recording their GCAMP6s fluorescence using a Prime BSI Express sCMOS camera (Teledyne Photometrics UK) and CoolLED *p*E-300 ultra light source via an Olympus 40× immersion objective and GFP filter cube (Chroma). The excitation waveband was 470–490 nm, applied at 2 Hz for 100 ms, and emission collected at 500–520 nm with a 495 nm long-pass filter. For analysis, ImageJ (v1.53c) was used to obtain mean fluorescence intensities over the image time series: active cell soma were selected manually as regions of interest (ROIs), and for each, the mean fluorescence values of a nearby background ROI was subtracted. Fluorescence intensity data and all metrics described below were analyzed using custom Python scripts. For each neuronal fluorescence trace, the following equation was used to generate ΔF/F:

$$\frac{\Delta F}{F} = \frac{F_t - F_{bg}}{F_0}$$

Where F_t is the mean somatic fluorescence measured at any time point, F_{bg} is the mean background fluorescence measured at the same time point, and F_0 is mean of the lower 8% of the ΔF fluorescence over the 26 s previous to that time point. 26 s was chosen as the GCAMP6s decay time constant τ (0.65 s) * 40.

Within a neuronal trace, an 'event' was registered where the ΔF/F trace exceeded 2 SD above the trace mean. An mSE was defined as comprising events from at least two cells with peak amplitudes occurring within 10 s of each other. Events occurring in other neurons were included in the mSE if they also peaked within 10 s of the previous event peak in the mSE. Event and mSE rates are presented as 'per cell, per hour', thereby controlling for the variation in the number of neurons being recorded in each brain slice. Above threshold activity in the *same* neuron was excluded if it reoccurred within 10 s of the previous event, as after reviewing typical *in vitro* activity, this was likely to represent above-threshold 'flicker', rather than a distinct calcium event of the type typically observed in these cells.

Within each experiment, a pre-drug baseline was obtained, followed a drug application period, and a wash period. All of these measurement periods, from which activity/synchronization rates were calculated, were 12 min each and followed/preceded by a 2-min gap to allow wash-in/out of compounds.

When determining shape properties of calcium events, the trace baseline value was calculated by taking the median value of all data points below a threshold of 0.5 SD above the trace mean. Qualitatively this method provided an excellent match with the actual baseline. Only neurons active in both the pre-drug and drug-applied measurement periods were included when evaluating drug-induced effects on shape, in order to avoid skewing the data in the event that different neurons begin or cease their activity in those periods. The mean of each of the shape properties was generated for each of these neurons, and each datapoint shown is a mean of means, representing one slice/experiment. A minimum of three neurons meeting these criteria was required to form a datapoint for that experiment. Half-width is defined as the width of the calcium event at 50% amplitude, from the calculated baseline to the peak. Rise time is the length of time taken to rise from 20% to 80% of maximum event amplitude, and decay time the reverse.

For the temporal correlation between neurons, experiments were performed over 40 min: mSEs involving <5 cells were excluded, and a minimum of three of these larger mSEs was required in order to calculate the correlation coefficient. Order within mSEs were calculated using the event peak time of each cell. In generating heatmaps and scatterplots, neurons were sorted in order of the mean event order over all mSEs occurring, making it possible to look for consistency in that order. Spearman correlation coefficients were calculated for scatterplots, representing the consistency in which ARN^{KISS} neurons reach peak amplitude in a similar position. High correlation coefficients indicate a consistent temporal order of firing whilst low correlation implies stochastic firing.

Brain slice electrophysiology and calcium imaging experiments

For current clamp experiments, patch pipettes were pulled from standard borosilicate glass (GC150F, Warner Instruments) to a resistance of 2.5–4 MΩ and filled with an intracellular solution composed of (mM): potassium gluconate 130; sodium gluconate 5, HEPES 10; $CaCl_2$ 1.5; sodium phosphocreatine 4; Mg-ATP 4; Na-GTP 0.3; pH 7.3; filtered at 0.2 μm. Experiments were performed as for normal calcium imaging but a single GCAMP-expressing kisspeptin neurons was patched and stimulated with ∼20–30 pA for 10–15 s in order to achieve the peak firing frequency of approximately 20 Hz. Co-incident activation of the patched neuron and other cells in the slice were evaluated as described in the results. For SFA experiments, three 10 s square wave 20 pA currents were applied before and during exposure to apamin and IbTX. To quantify the degree of SFA, we divided the inter-spike interval (ISI) following spike 1 by the ISI following spike 10, with a value of 1 representing no SFA and a value of 0.5 representing a 50% reduction.

Stereotaxic surgery and injections

Adult mice (8–14 weeks old) were anesthetized with 2% Isoflurane, given meloxicam (5 mg/kg, s.c.), buprenorphine (0.05 mg/kg, s.c.) and dexamethasone (10 mg/kg, s.c.) and placed on a stereotaxic apparatus. A Hamilton syringe with a 25-gauge needle was used to perform unilateral injections into the ARN. The needle was lowered into place over 3 min and left *in situ* for 3 min before the injection was made. One microliter of AAV9-CAG-FLEX-GCaMP6s-WPRE-SV40 (1.3×10^{13} GC/mL, University of Pennsylvania Vector Core) was injected into the ARN (2.0 mm posterior to bregma, 0.3mm lateral to midline, 5.9 mm deep from brain surface) at a rate of 100 nL/min with the needles left *in situ* for 10 min before being withdrawn. Meloxicam (5 mg/kg) was administered orally for post-operative pain relief. For fiber photometry experiments, mice received a unilateral injection of AAV-GCaMP6 into the ARN followed by implantation of an indwelling "fluidic" optical fiber (400 μm diameter, 0.48 NA; 485 μm OD for guiding tube, Doric Lenses, QC, Canada), positioned directly above the mid-caudal ARN using the same coordinates for the AAV injections. For GRIN lens miniscope experiments, AAV-GCaMP6 injection was followed by implantation of a GRIN lens (500 μm in diameter, 8.4mm in length, Grintech, Germany), into the ARN using the same coordinates except for the DV coordinates being 200 μm above the injected site. After 5 weeks, a baseplate was put in place above the GRIN lens for attachment of miniature microscope (UCLA open-source Miniscope, V2) under isoflurane anesthesia. Following one week of surgery, all animals were handled daily and habituated to recording setup for at least three weeks.

GCaMP6 GRIN lens miniscope imaging *in vivo*

A single-photon epifluorescence microscope (UCLA open source, https://github.com/Aharoni-Lab/Miniscope-v4) was used as described previously.[60] Images were acquired at 30 frames per second and recorded as uncompressed.avi files. For analysis,

MiniscopeAnalysis package (Sylvain Williams Lab, McGill University) written in MATLAB was used to process raw videos and extract relevant data. NormCorre and CNMFE were used for motion correction and signal extraction.

Extracted data were down sampled to 10 fps and spatial footprints of cells manually inspected with footprints >25px considered to represent signals from cell bodies. All raw traces were normalized to z-scores for quantitative analyses. An SE was identified as an increase in the fluorescence above 1 SD in more than 5 cells. The peak of an SE was identified with MinPeak algorithm and all signals from cells contributing to an SE were averaged to determine the mean duration, time taken to rise and recover to the half-width full maximum and the interpeak intervals. The peak times of individual cells were measured to determine whether neurons peak in a predictable order in an on-going series of SEs. The mean order of cells according to their peak times in all of the SEs within a recording was used to rank the cell ID and the order of peak was plotted against the ranked cell ID. The Spearman correlation coefficient was calculated from scatterplots to determine the likelihood of cells firing in an order. To assess any emergent activity in neurons prior to the SE, the linear slope of two blocks of 3-min baseline activity (1.5–4.5min and 4.5 to 7.5min prior to the peak) were determined in each cell. A change in slope >5% was considered to exhibit "baseline ramping". A Fast Fourier Transform algorithm was applied to each block to qualitatively examine if changes in frequency domain occur during these periods.

GCaMP6 fiber photometry

Fiber photometry was performed using a custom-built fiber photometry system using Doric components (Doric Lenses, QC, Canada) and National Instrument data acquisition board (National Instruments, TX, USA) based on a previous design.[8,62] Blue (465–490 nm) and violet (405 nm) LED lights, sinusoidally modulated at 531 and 211 Hz respectively, were focused onto a single 400 μm-diameter fiber optic linked to the mouse. The light intensity at the tip of the fiber was set between 30 and 80 μW. Fluorescence emitted from the brain was collected by the same fiber, passed through a 500–550 nm emission filter, and focused onto a Doric fluorescence detector. The emissions were collected at 10Hz in a scheduled 5s on/10s off mode and two GCaMP6 emissions were recovered by demodulating the 465 nm signals and 405 nm signals. Intra-ARN microinfusions were coupled with GCaMP fiber photometry by using custom-designed optic fibers incorporating an infusion cannula and port in the head mount (Doric Lens, QC, Canada). After recording GCaMP activity for 60 min, 1μL of either TTX (5 μM), CNQX (100 μM) +AP5 (500 μM), the tachykinin receptor antagonist cocktail (GR94800 (10 μM), SDZ NKT 343 (10 μM), SB222200 (30 μM), norBNI (100 μM) or vehicle were infused at a rate of 100 nL/min through the injection cannula connected to a Nanofil microinjection pump via 25-gauge polyethylene tubing. Antagonists were used at 5-10-fold higher than the effective dose in brain slice studies. Photometry recordings continued throughout the infusion period for a total duration of 120 min. Each animal received three different compounds or vehicle in random order with a 10-day interval between each.

Analysis was performed in MATLAB with the msbackadj algorithm used to correct for baseline shift, with the time window of 900 s. The signals (465-405) were then normalized to z-scores for quantitative analyses. The Findpeaks algorithm was used to detect SEs, and the duration and the time of SE to the half-width full maximum was determined. To examine the effect of pharmacological agents on SE occurrence, the mean *Z* score of the fluorescent signal 40 min before, 10 min during, and 40 min post drug infusion period was averaged.

Immunohistochemistry

Adult Kiss1-Cre,Ai126D mice were given a lethal overdose of pentobarbital (3mg/100μL, i.p.) and perfused transcardially with 4% paraformaldehyde. Brains were processed for dual GFP and kisspeptin immunofluorescence as reported previously.[6] Primary antisera were chicken anti-GFP (1:5,000, Aves Labs) and sheep anti-kisspeptin-52 (1:1,000, AC053; gift from Alain Caraty) and followed by biotinylated donkey anti-sheep immunoglobulin (1:1,000, Jackson Immunoresearch) and AlexaFluor 488-conjugated goat anti-chicken (1:1,000) and AlexaFluor 568-conjugated Streptavidin (1:400, Molecular Probes). Imaging was undertaken using a Leica SP8 Laser Scanning Confocal Microscope (Leica Microsystems) at the Cambridge Advanced Imaging Center and analyzed using ImageJ.

Receptor and channel antagonists

NorBNI, CNQX, D-AP5, and NKB were obtained from Sigma-Aldrich UK, and SDZ-NKT 343, GR 94800, and SB 222200, apamin, iberiotoxin, cyclothiazide and TTX obtained from Tocris (Bio-Techne) UK. Standard laboratory salts were purchased from Fisher Scientific UK.

QUANTIFICATION AND STATISTICAL ANALYSES

Statistical analyses were performed in Prism 9 (GraphPad software Inc.). All values given in the text and within figures are mean ± SEM, and significance is defined as $p < 0.05$*, $p < 0.01$**, or $p < 0.001$***. For *in vitro* studies, the Shapiro-Wilk normality test was used to assess the distribution of unpaired datasets, and residuals for paired datasets: nonparametric tests were applied where data did not pass the normality test. All analyses were two-tailed, and all experiments were replicated in a minimum of five animals per group, with n representing the number of brain slices. Outliers were detected using Grubbs' test and, across all datasets, only one outlier was removed (Shape metric data using TKR antagonists in gonadectomised males). For *in vivo* experiments, two-way mixed ANOVA was used to compare the endogenous and norBNI-evoked SE, followed by Sidak's post-hoc test. Chi-squared test was used to compare the probability of SE occurrence during drug infusions. One-way RM ANOVA was applied to compare the mean fluorescence signal before, during and after the drug infusion, followed by Dunnett's post-hoc test. N represents the number of animals.

Cell Reports, Volume *42*

Supplemental information

Mechanism of kisspeptin neuron synchronization for pulsatile hormone secretion in male mice

Su Young Han, Paul G. Morris, Jae-Chang Kim, Santosh Guru, Maria Pardo-Navarro, Shel-Hwa Yeo, H. James McQuillan, and Allan E. Herbison

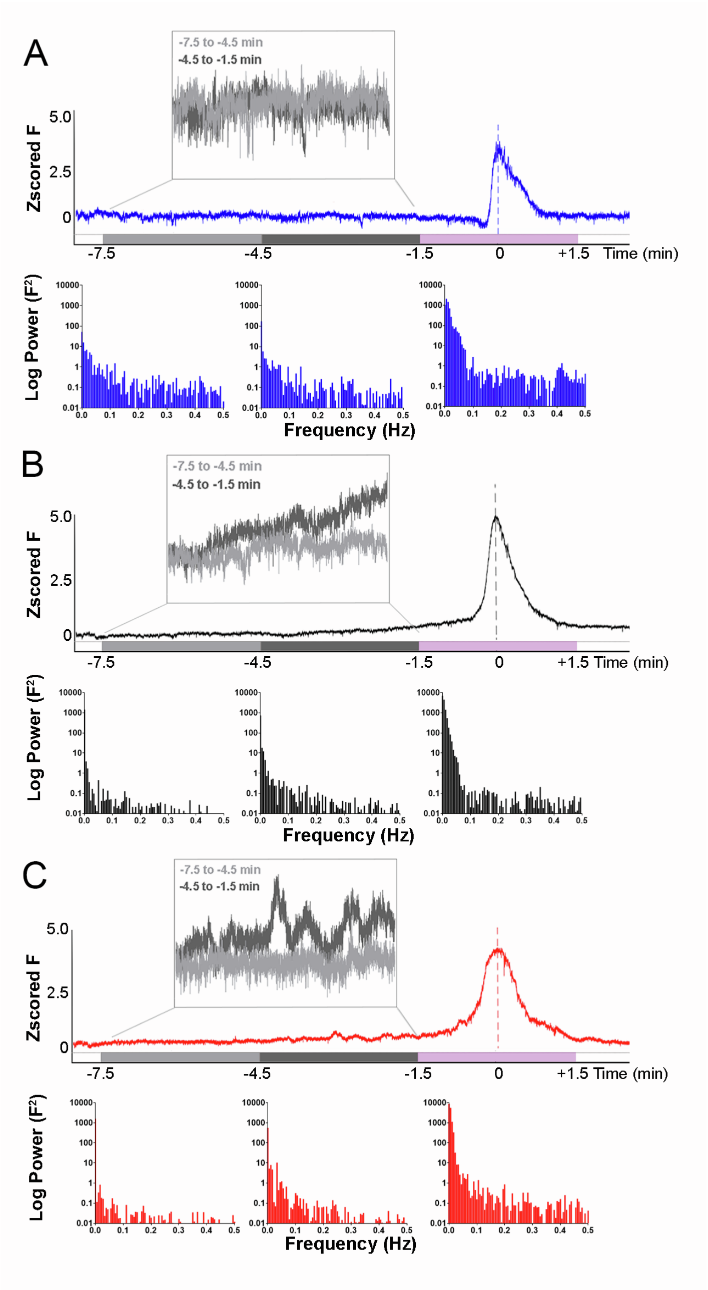

Suppl. Figure 1. *"Ramping" activity of individual ARN^{KISS} neurons in vivo*. **A-C,** GCaMP6 traces from three individual ARN^{KISS} neurons showing different patterns of activity leading up to a synchronization. Each section shows GCaMP6 levels (Z-scored F) in the 7.5 min period leading up to and including the abrupt increase in activity. The inset above shows overlapping fluorescence traces for the early 7.5-4.5 min (light grey) and close 4.5-1.5 (dark grey) min periods prior to a synchronization peak. Below, the power spectral density plot (log Power F^2) of slow frequency domain between 0-0.5 Hz for each 3 min bin showing the relative amounts of activity in the different frequency domains. In A, the activity spectra are very similar in the two blocks (7.5-4.5 and 4.5-1.5 min prior to synchronization) whereas for B and C there is an increase in low frequency activity during the -4.5 to -1.5 min blocks.

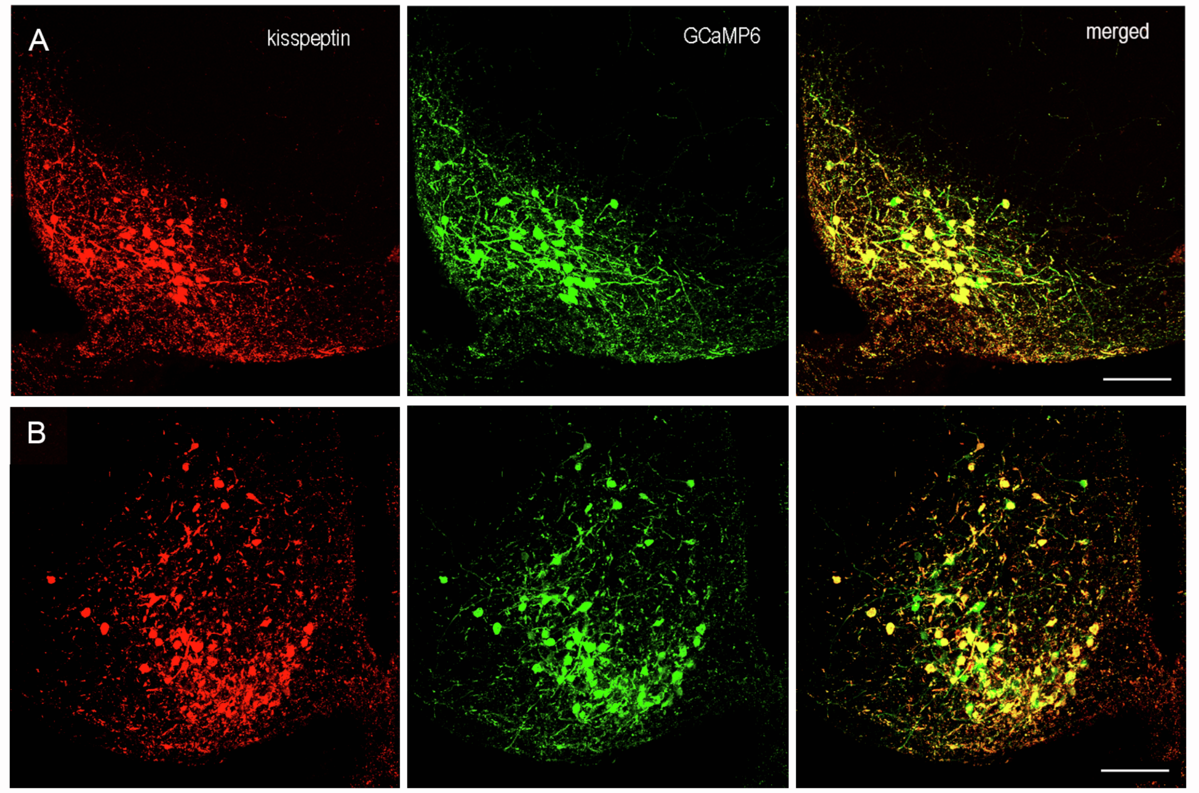

Suppl. Figure 2. *Characterization of Kiss1-Cre,Ai126D mice*. Dual label immunofluorescence at mid- (A) and caudal (B) coronal levels of the ARN showing the kisspeptin (red), GCaMP6 (green) and overlap of the two signals. Scale bars represent 100 µm.

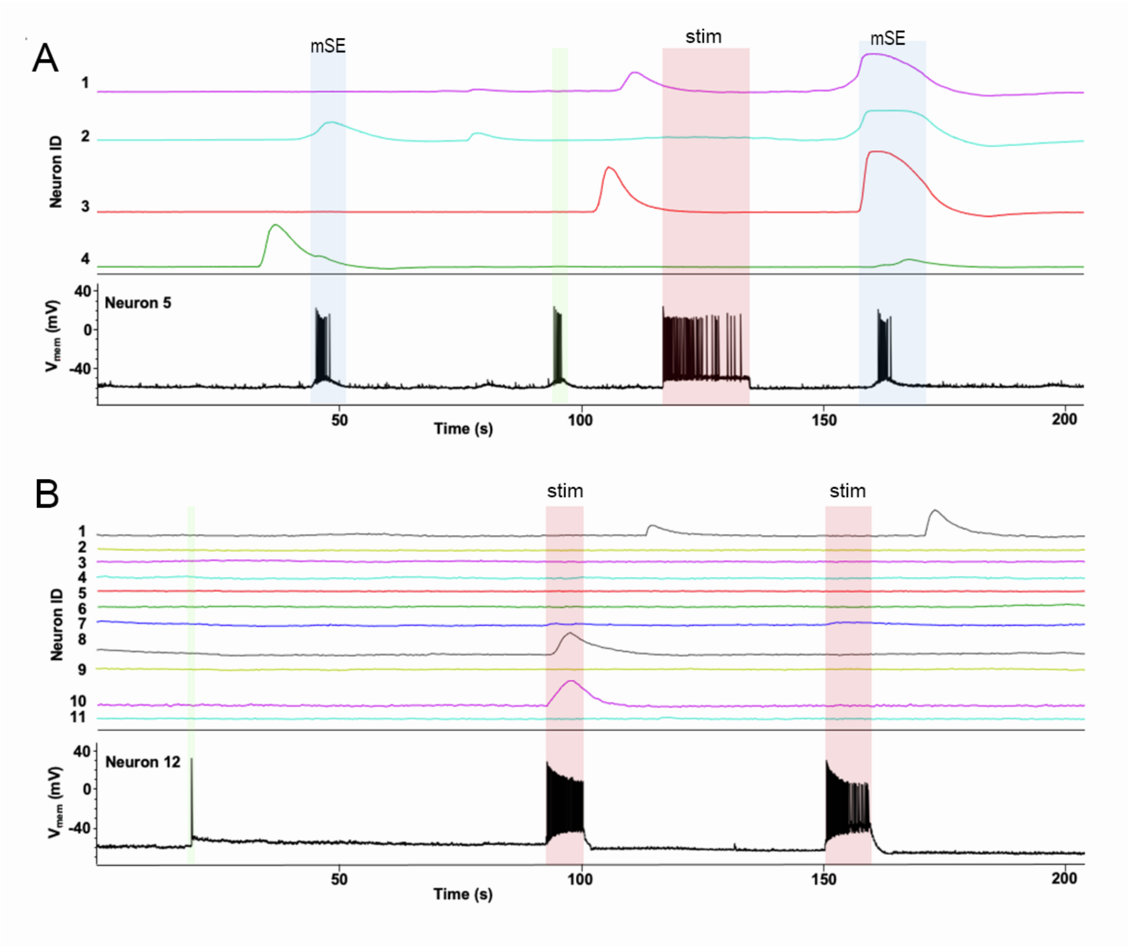

Suppl. Figure 3. *Effects of electrical activation of one ARN^{KISS} neuron on GCaMP activity of other ARN^{KISS} neurons in the brain slice.* **A,** GCaMP traces from 4 ARN^{KISS} neurons in the same slice as Neuron #5 (bottom trace) that was patched. Neuron #5 has three spontaneous episodes of burst firing with two associated with GCaMP increments other neurons (representing mSEs, blue) and one alone (green). However, artificial activation of Neuron #5 (pink) has no immediate effects on any of the other recorded cells. **B,** GCaMP traces from 11 neurons and a patched cell Neuron #12. Activation of firing in neuron #12 results in synchronized increases in GCaMP signal in neuron #8 and #10 on the first occasion but not subsequently.

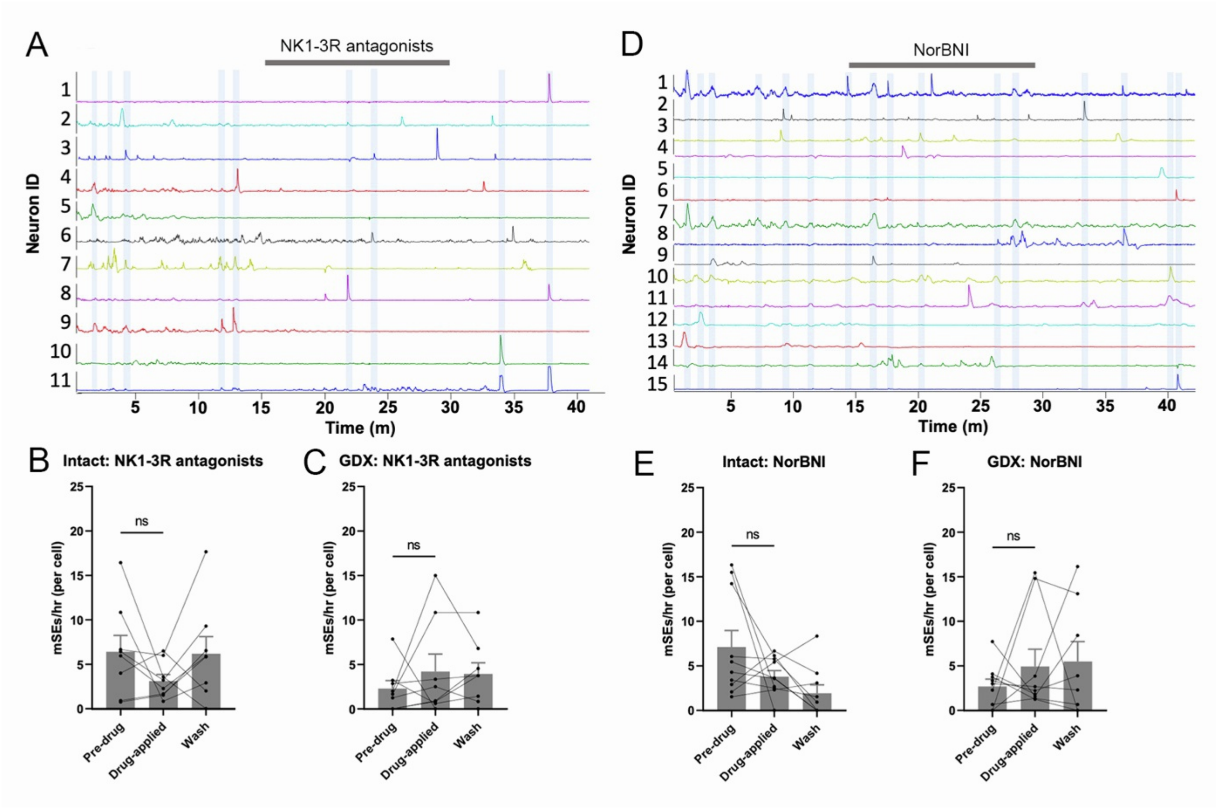

Suppl. Figure 4. *Tachykinin and dynorphin signaling are not essential for ARN^{KISS} neuron synchronization in vitro.* **A&D,** Recordings from brain slices showing the effect of 15 min exposure to the tachykinin receptor antagonist cocktail (1 μM SDZ-NKT 343, 1 μM GR94800, 3 μM SB 222200, intact male) or NorBNI (10 μM; GDX male) on GCaMP fluorescence recorded from 11 and 15 kisspeptin neurons. mSEs are indicated by blue shading. **B&C,** Histograms showing the individual data points and mean (+SEM) mSE rate for the pre-drug, drug-applied and wash periods in response to the tachykinin receptor antagonist cocktail in intact and GDX male mice brain slices. **E&F,** Histograms showing the individual data points and mean (+SEM) mSE rate for the pre-drug, drug-applied and wash periods in response to NorBNI in intact and GDX male mice brain slices.

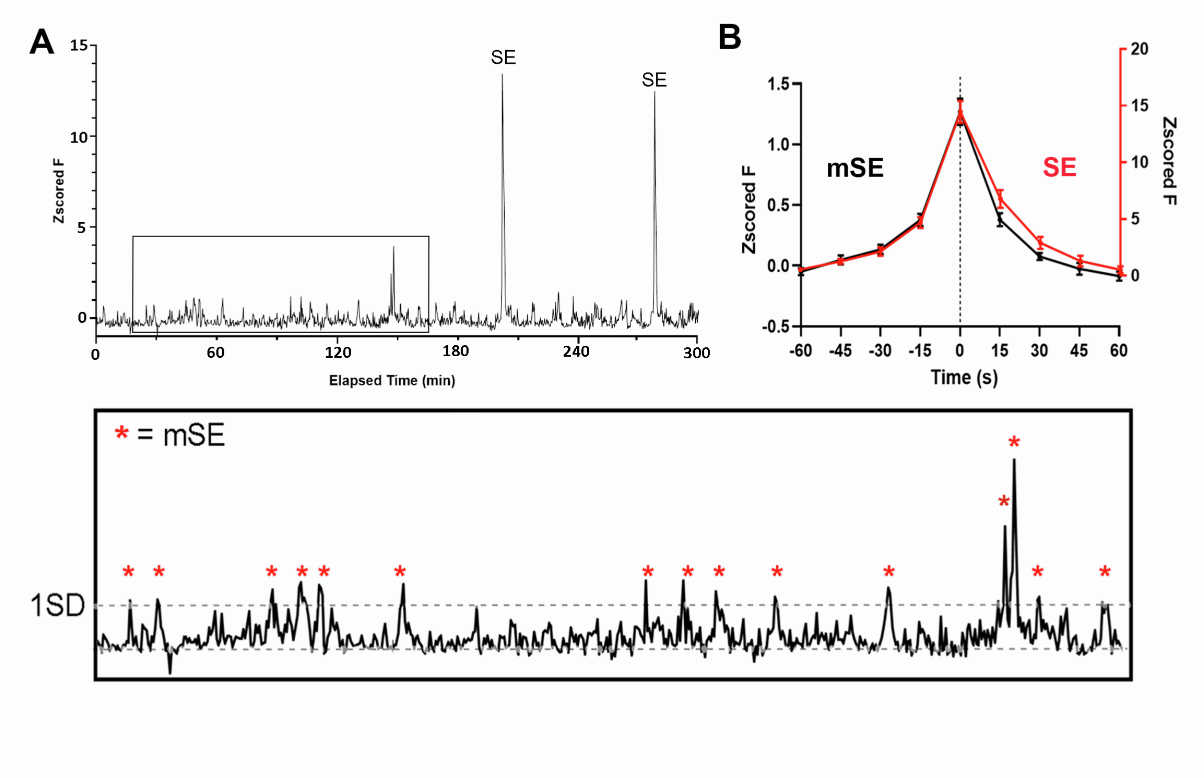

Suppl. Figure 5. *Low amplitude miniature SEs occur in vivo.* **A,** GCaMP fiber photometry recording from an intact male mouse showing the occurrence of two SEs. Baseline recording is expanded below to show frequent low-amplitude miniature synchronization events (mSEs, asterisks). Baseline and 1 standard deviation (SD) from mean is indicated by the dotted lines. **B,** Mean±SEM amplitude normalized profiles of SEs (red, 9 events) and mSEs (black, 269 events) recorded from intact male mice (N=5).

Suppl. Table 1. No changes occur in the dynamics of mSEs in the presence of antagonists for ionotropic glutamate receptors (CNQX + AP5), tachykinin receptors (TKR) or kappa opioid receptors (NorBNI).

	**Intact male**				**GDX male**			
	**Half width (s)**	**Rise time (s)**	**Decay time (s)**		**Half width (s)**	**Rise time (s)**	**Decay time (s)**	
**CNQX + AP5**				n=6				n=6
Pre-drug	13.8 ± 1.31	4.57 ± 0.436	6.52 ± 0.946		14.1 ± 1.11	4.51 ± 0.711	5.67 ± 0.523	
Drug	12.6 ± 1.61	3.65 ± 0.560	7.59 ± 0.704		20.4 ± 2.56	7.88 ± 2.28	7.44 ± 0.817	
**TKR antagonists**				n=7				n=5
Pre-drug	12.0 ± 1.84	4.00 ± 0.927	6.27 ± 0.420		13.6 ± 2.40	3.53 ± 0.730	6.73 ± 1.30	
Drug	10.1 ± 0.697	2.84 ± 0.584	6.65 ± 0.506		15.5 ± 2.64	5.23 ± 1.14	7.04 ± 0.347	
**NorBNI**				n=10				n=8
Pre-drug	12.9 ± 1.04	3.69 ± 0.288	6.43 ± 0.307		18.2 ± 3.23	5.51 ± 1.30	7.18 ± 0.795	
Drug	12.8 ± 1.15	4.18 ± 0.705	6.79 ± 0.624		13.8 ± 1.61	4.28 ± 0.842	6.12 ± 0.502	
**Cyclothiazide**				n=8				
Pre-drug	9.92 ± 0.89	3.56 ± 0.544	6.34 ± 0.423					
Drug	10.83 ± 0.75	3.01 ± 0.385	6.79 ± 0.624					
